# 3D Fabrication with Integration Molding of a Graphene Oxide/Polycaprolactone Nanoscaffold for Neurite Regeneration and Angiogenesis

**DOI:** 10.1002/advs.201700499

**Published:** 2018-01-26

**Authors:** Yun Qian, Jialin Song, Xiaotian Zhao, Wei Chen, Yuanming Ouyang, Weien Yuan, Cunyi Fan

**Affiliations:** ^1^ Shanghai Jiao Tong University Affiliated Sixth People's Hospital 600 Yishan Road Shanghai 200233 China; ^2^ Shanghai Sixth People's Hospital East Campus Shanghai University of Medicine and Health Shanghai 201306 China; ^3^ School of Pharmacy Shanghai Jiao Tong University No. 800 Dongchuan Road Shanghai 200240 China

**Keywords:** graphene oxide, nerve conduits, peripheral nerve injuries, signaling pathways, vascular endothelial growth factor

## Abstract

Treating peripheral nerve injury faces major challenges and may benefit from bioactive scaffolds due to the limited autograft resources. Graphene oxide (GO) has emerged as a promising nanomaterial with excellent physical and chemical properties. GO has functional groups that confer biocompatibility that is better than that of graphene. Here, GO/polycaprolactone (PCL) nanoscaffolds are fabricated using an integration molding method. The nanoscaffolds exhibit many merits, including even GO nanoparticle distribution, macroporous structure, and strong mechanical support. Additionally, the process enables excellent quality control. In vitro studies confirm the advantages of the GO/PCL nanoscaffolds in terms of Schwann cell proliferation, viability, and attachment, as well as neural characteristics maintenance. This is the first study to evaluate the in vivo performance of GO‐based nanoscaffolds in this context. GO release and PCL biodegradation is analyzed after long‐term in vivo study. It is also found that the GO/PCL nerve guidance conduit could successfully repair a 15 mm sciatic nerve defect. The pro‐angiogenic characteristic of GO is evaluated in vivo using immunohistochemistry. In addition, the AKT‐endothelial nitric oxide synthase (eNOS)‐vascular endothelial growth factor (VEGF) signaling pathway might play a major role in the angiogenic process. These findings demonstrate that the GO/PCL nanoscaffold efficiently promotes functional and morphological recovery in peripheral nerve regeneration, indicating its promise for tissue engineering applications.

## 1 Introduction

Although peripheral nerves exhibit some self‐healing potential after mild and moderate trauma as they spontaneously start new axons sprouting after injury,^1^ successful reinnervation cannot be achieved, especially for long‐range nerve defects and it calls for implantation of a nerve graft to bridge the gap.^2^ Conventional surgical treatment has plateaued because the gold‐standard protocol‐autologous nerve transplantation causes unavoidable secondary damage to the donor site.^3^ Tissue engineering and bioactive materials are ideal alternatives for use in the circulatory, digestive, respiratory and nervous systems.^4^, ^5^, ^6^, ^7^ Many studies have reported that nerve guidance conduits (NGCs) exhibit extensive capabilities for repairing large nerve defects in the peripheral nervous system.^8^, ^9^, ^10^ NGCs are expected to direct cell migration in a targeted manner with their physical and chemical characteristics and to facilitate cell proliferation and differentiation. Synthetic materials such as polycaprolactone (PCL) possess several advantages such as biodegradability, nontoxicity, and structural stability. PCL has been tested in various applications, and has been shown to positively influence cardiovascular, nervous and soft tissues.^11^, ^12^, ^13^

Another important aspect of nerve tissue engineering is emphasized on inherent electrical excitability of nerve cells. Electrical stimulation can promote neurite extension and axonal regrowth. Conductive scaffolds have better electrical conductivity, biocompatibility and lipophilicity for cell adhesion.^14^, ^15^, ^16^ Among them, graphene is an extremely important nanomaterial due to its exceptional physical and chemical properties.^17^, ^18^ Moreover, graphene can interact with biomolecules such as proteins, polypeptides and nucleic acids in regenerative medicine.^19^ Graphene oxide (GO) is an extremely oxidized graphene derivative with a 2D structure consisting of a single layer with multiple functional groups.^20^ It exhibits amphiphilic characteristics because of the presence of epoxide, hydroxyl and carboxylic acid functional groups in the same plane.^21^ It has been previously reported that the application of potassium permanganate and sulfuric acid in graphite has previously been reported to facilitate GO nanosheets separations.^22^ Unmodified graphene areas produce a large aromatic interface of free π electrons and the unique physical and chemical nature of this interface allows GO to interact with proteins, peptides, and DNA.^23^ Aligned poly‐l‐lactide scaffolds coated with GO have demonstrated an excellent ability to mediate neurite growth and differentiation.^24^ In addition, GO/polyacrylamide composite hydrogels with 0.4% GO content can improve the attachment and proliferation of Schwann cells.^25^ GO and PCL can also be successfully combined to fabricate biocompatible nanofibrous scaffolds. Furthermore, the addition of moderate amounts of GO to PCL significantly strengthened the attachment and proliferation of mesenchymal stem cells.^26^

In addition, researchers exert external electrical stimulation to graphene and other conductive materials and improve nerve regeneration. Heo et al. fabricated graphene/polyethylene terephthalate film to induce nerve differentiation under electrical stimulation. They effectively strengthened cell‐cell communication, which might result from changes in the intercellular coupling with endogenous cytoskeletal proteins.^27^ Song et al. fabricated polypyrrole/PLCL (Poly (l‐lactic acid caprolactone)) conduit and used it to influence PC12 cells and dorsal root ganglion in vitro and peripheral nerve repair in vivo under electrical stimulation.^28^ Therefore, we wonder whether GO/PCL scaffold alone can also improve peripheral nerve regeneration and why it is capable of doing so. Furthermore, we assume that GO/PCL scaffold might conduct the bioelectricity within peripheral nerves to accelerate nerve repair process without external electrical stimulation.

Apart from the excellent electrical conductivity, biocompatibility and physical adsorption of GO, recent studies have made breakthroughs in elucidating pioneering roles of GO in angiogenesis. Angiogenesis is a basic process in tissue regeneration. Many strategies of nerve tissue engineering emphasize on mimicking native nerve characteristics including architecture, and protein composition. It serves as a bridge to direct reinnervation and provide necessary nutrition for nerve regeneration. GO can induce angiogenesis and contribute to nutrient formation and transportation in bone regeneration.^29^ Low concentrations of GO can be pro‐angiogenic by regulating the intracellular expression of reactive oxygen species (ROS) and reactive nitrogen species (RNS).^30^, ^31^ We also evaluate this important property of GO after long‐term nerve restoration in vivo and reveal the underlying mechanism in this study.

In this study, we used a novel integration molding method to fabricate GO/PCL nanoscaffolds. Biocompatibility, cellular proliferation, and neural differentiation were evaluated using rat Schwann 96 cells (RSCs), a very commonly studied cell type in peripheral nerve regeneration experiments in vitro.^32^ This was the first study to extensively evaluate the long‐term performance of GO‐based nanoscaffolds in peripheral nerve restoration. In vivo, the GO/PCL NGCs could efficiently heal a 15 mm sciatic nerve defect in a Sprague Dawley rat model by 18 weeks after injury. We also validated the influence of these innovative GO/PCL conduits on angiogenesis and explored the underlying mechanism.

## 2 Results and Discussion

**Figure 1:**
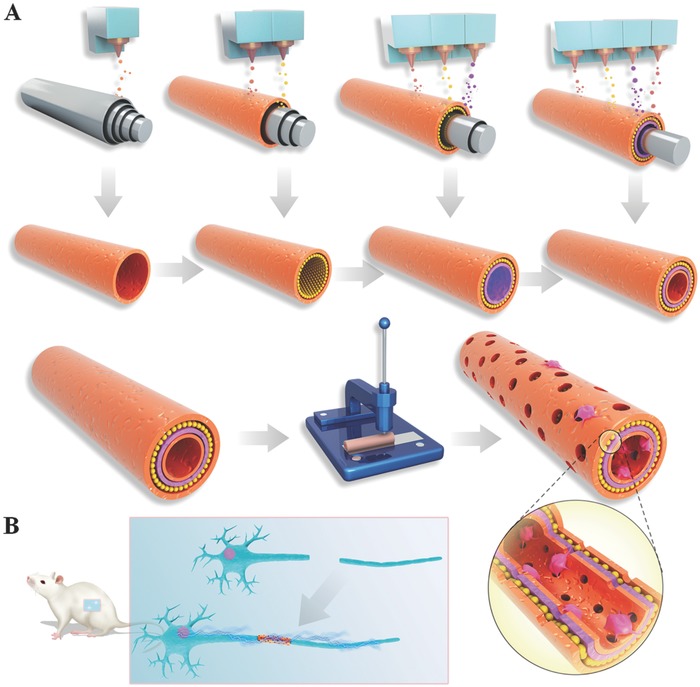
Schematic illustration of GO/PCL nanoscaffold fabrication by the integration molding method A) and NGC implantation in the rat model B). We prepared a tubular mold that included four tubes of concentric circles. Two complex tubes are located inside the inner‐most tube and the outer‐most tube, forming a concentric circle structure. A GO and PCL mixture was injected into the space between the outer‐most layer and the second outer‐most layer. After solidifying, the second outer‐most layer was removed, and the GO/PCL mixture was injected into the space between the second outer‐most layer and the third outer‐most layer. The same procedure was repeated again between the third outer‐most layer and the inner‐most layer. Finally, a 3D printer was used to create multiple aligned pores in the surface of the GO/PCL conduit.

In this study, an integration molding method was used to fabricate GO/PCL nerve nanoscaffolds (**Figure** **1**). A tubular mold consisting of four tubes of concentric circles had been previously prepared. Two complex tubes were located between the inner tube and outer‐most tubes to form a concentric circle structure. A mixed solution of GO and PCL was injected into the space between the outer‐most and second outer‐most layers. After solidifying, the second outer‐most layer was removed, and the GO/PCL solution was injected into the space between the second and third outer‐most layers. The procedure was repeated a third time between the third outer‐most layer and the inner‐most layer. Finally, a 3D printer was used to create multiple aligned pores in the surface of the GO/PCL conduit. The multi‐layered structure strengthened the mechanical properties of the nerve conduit. In addition, the several porous layers facilitated biodegradation and optimized the long‐term in vivo performance of the NGC because the macropores between the different layers increased the possibility of internal contact with body fluid. Another major challenge in nerve tissue engineering is how to treat long‐range nerve defect over a long‐term regeneration. A strategy to do this is a controlled release system and a slow but steady biodegradation substrate material. In this study, the 3D printing enabled us to fabricate the conduit with a bottom‐up style, which indicates integrated multi‐layered fabrication. In addition, the printer also permitted digital control of mixed solution injection, and this resulted in an even distribution of different biomaterials in the conduit. Furthermore, the 3D printing enabled us to create a conduit with certain volume of different elements and to fabricate it from any angle, position, or plane. With high resolution, 3D printing could better improve RSC proliferation, attachment and neural expression.

**Figure 2:**
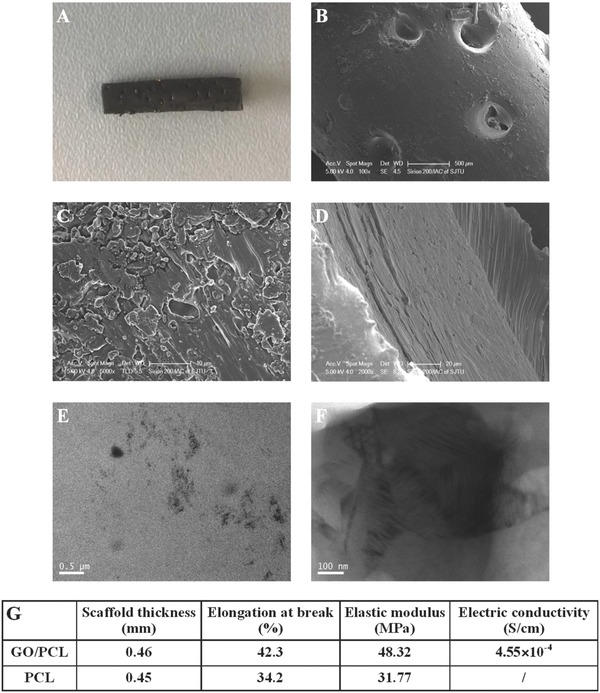
Characterization of the GO/PCL NGC. Optical images of the GO/PCL NGC A). SEM images showing the nanoporous structure of the GO/PCL NGC B,C) and the multilayered structure of an ultra‐thin section D). TEM images showing the uniform distribution of GO nanoparticles in the PCL scaffolds E,F). Mechanical and electrical properties, i.e., scaffold thickness, elongation at break, elastic modulus, and electrical conductivity of the GO/PCL and PCL scaffolds G).

We characterized the GO/PCL nanoscaffold morphology by scanning electron microscopy (SEM) and optical imaging (Sirion 200/Instrumental Analysis Center (IAC), **Figure** **2**). The nanoparticles and the stiff surface are shown at various magnifications. The macroporous structure could be identified at a lower magnification, revealing a clear and oriented alignment. The presence and distribution of GO nanoparticles in the tubular PCL structure were evaluated by transmission electron microscopy (TEM). The GO was confirmed to have an oriented distribution in the cross‐section of the GO/PCL nanoscaffolds (Figure 2). The diameter of the pores was just several micrometers due to the adjustable porosity of the 3D printer. The multiple aligned pores in the conduit enable exchanges of water, oxygen and other nutrients. At the same time, it prevents alien cells such as fibroblasts from entering the conduit and interfering with normal and functional nerve regrowth.

We further evaluated two mechanical properties of the 3D conduit, the scaffold thickness and the elastic modulus. The scaffold thickness was similar for both materials. Therefore, the addition of GO did not influence this property. However, the average elastic modulus of the GO/PCL conduit was 48.32 MPa, in contrast to 31.77 MPa for the PCL conduit. The elongation at break results also indicated that the addition of GO improved the mechanical strength of the nanoscaffolds. The reason for the improved elastic modulus and increased elongation at break of the GO/PCL nanoscaffolds is that the continuity of the PCL matrix interferes with the even distribution of the GO nanoparticles. In addition, GO can interact with the molecular structures of PCL, leading to increased intermolecular strength,^33^ which was confirmed by the transmission FTIR spectroscopy in this study (Figure S1, Supporting Information). The mechanical test confirmed that the porous 3D GO/PCL conduit could maintain its tubular structure and allow nerve growth. The electrical conductivity of different scaffolds was also evaluated. The GO/PCL conduit displayed a relatively high conductivity of 4.55 × 10^−4^ S/cm, while the electrical conductivity of the PCL conduit was 0 S/cm. According to the above results, the GO/PCL NGC exhibited excellent mechanical and topological properties, including a multi‐layered design, ideal rigidity and flexibility, microporosity for the free exchange of nutrients, relatively high electrical conductivity, and even GO nanoparticle distribution for efficient drug delivery. The mechanical performance was improved by 3D printing multi‐layered fabrication in comparison with non‐layered counterparts (Table S1).

**Figure 3:**
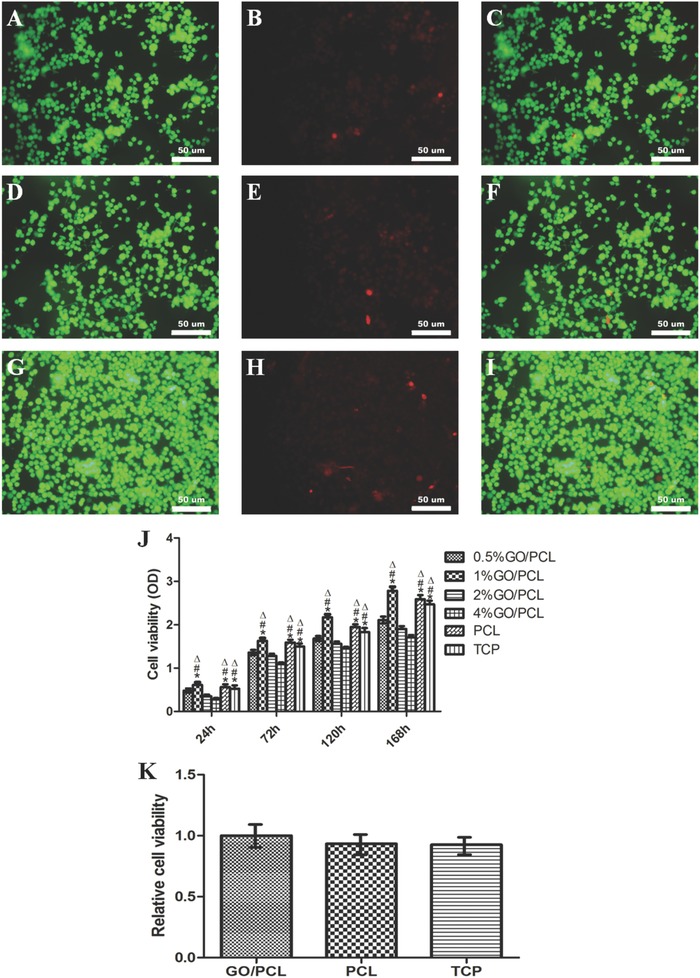
Cell viability as assayed by LIVE/DEAD cell staining on GO/PCL scaffolds A–C), PCL scaffolds D–F) and TCP G–I). Live cells (green fluorescence, A, D, and G). Dead cells (red fluorescence, B, E, and H). Merged images (C, F, and I). The scale bar is 50 µm. CCK‐8 assay for RSCs cultured on GO/PCL scaffolds with different concentrations of GO, PCL scaffolds and TCP at 24, 72, 120, and 168 h J). *P < 0.05 compared with 0.5% GO/PCL. ^#^ *P* < 0.05compared with 2% GO/PCL. Δ*P* < 0.05 compared with 4% GO/PCL. Relative cell viability was evaluated by the LIVE/DEAD cell staining for 1% GO/PCL scaffolds, PCL scaffolds and TCP K).

To determine the optimal GO percentage in the PCL scaffolds, we cultured Schwann cells on 0.5% GO/PCL, 1% GO/PCL, 2% GO/PCL, 4% GO/PCL, and PCL scaffolds in 24‐well plates for 1, 3, 5, and 7 days. The medium was replaced every two days. Twenty microliters of CCK‐8 solution was added to 200 µL of medium in each well, and cells were further cultured in a 5% CO_2_ incubator for 4 hours. Then, 100 µL of medium from each well was transferred to a new 96‐well plate and the absorbance at a wavelength of 450 nm was determined using a multifunctional microplate reader (Thermo 3001, Thermo Fischer Scientific, USA). A tissue culture plate (TCP) was used as a control. This procedure was repeated for three independent samples for each scaffold. The results showed greater cell proliferation on the 1% GO/PCL scaffold than on the other scaffolds (*p* < 0.05) and that this proliferation was similar to that on PCL and TCP (*p* > 0.05) (**Figure** **3**). The fate of cell proliferation is determined by the nanoscaffold. The optimal amount of GO in the PCL scaffold would promote the greatest extent of RSC proliferation, which is important for further evaluating cell attachment, viability, and neural expression. Therefore, the 1% GO/PCL scaffold was selected for further evaluation. We found that GO was relatively biocompatible at a low level. A recent study on the influence of GO on cell proliferation indicated that the application of more than 2% GO in cell culture could lead to significant cytotoxicity.^34^ This finding was consistent with our study, and we found that GO/PCL could be a biocompatible material at relatively low concentrations of GO.

To verify the viability of RSCs on the different nanoscaffolds, cell viability was assayed using a LIVE/DEAD cell kit for mammalian cells (Invitrogen) according to standard protocols. The images displayed high percentages of live cells and negligible differences were found in cell viability among the GO/PCL, PCL, and TCP groups (Figure 3).

**Figure 4:**
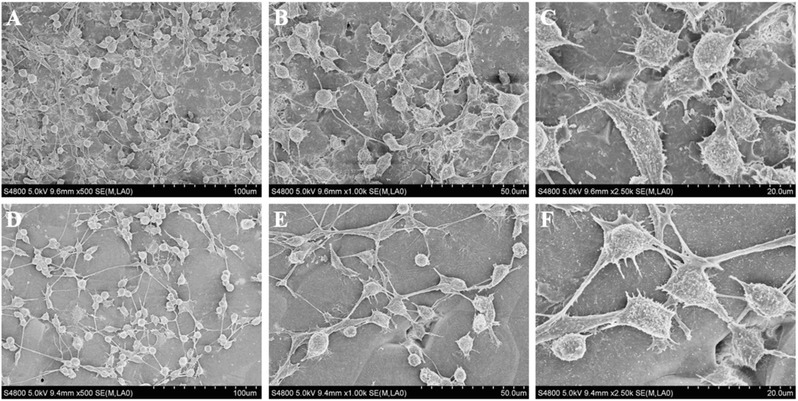
SEM images showing RSC morphology on GO/PCL and PCL scaffolds. RSCs were cultured on GO/PCL, and PCL nanoscaffolds for 4 d before observation. GO/PCL scaffold A–C). PCL scaffold D–F). The scale bars are 100 µm A,D), 50 µm B,E), and 20 µm C,F), respectively.

Cell attachment is important for ideal cell viability. SEM, immunofluorescence and real‐time quantitative PCR (qPCR) were adopted to test cell attachment and morphology on the different nanoscaffolds. After being cultured on the different scaffolds for 4 d, the RSCs were ready for SEM observation. The SEM images revealed that most of the scaffolds were covered with cells exhibiting normal structures and extended protuberances (**Figure** **4**).

**Figure 5:**
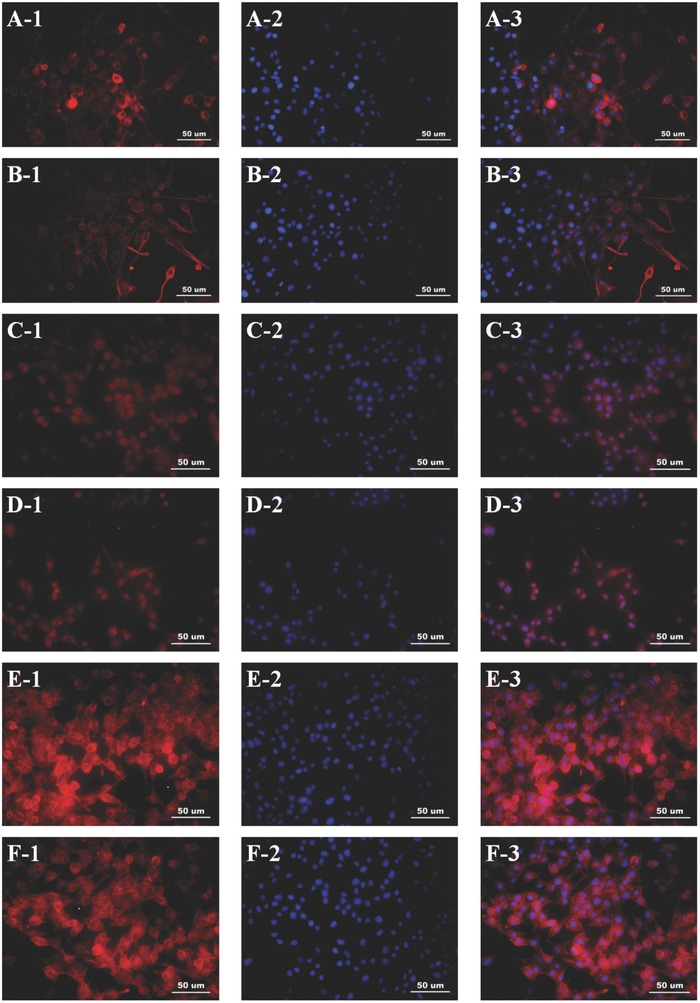
Immunofluorescence staining for Tuj1 A,B), Ki67 C,D), and phalloidin E,F). All samples were washed three times, fixed with 4% paraformaldehyde for 20 min at 25 °C and blocked with BSA overnight. DAPI staining appears blue. GO/PCL scaffolds A1–A3, C1–C3, and E1–E3). PCL scaffolds B1–B3, D1–D3, and F1–F3). The scale bar is 50 µm.

Cell morphology and attachment were evaluated by staining actin cytoskeleton with phalloidin. Immunofluorescence showed the attachment and morphology of the cells on the GO/PCL and PCL scaffolds. The cells exhibited normal spindle‐like shapes (**Figure** **5**). This result further demonstrated the functional bioactive environment provided by the GO/PCL nanoscaffolds.

**Figure 6:**
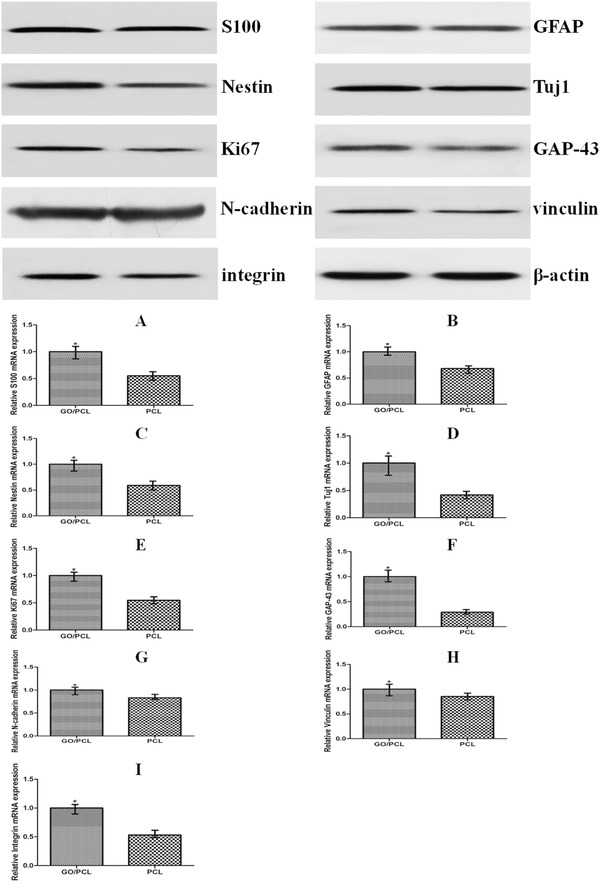
Western blot and qPCR results for S100 A), GFAP B), nestin C), Tuj1 D), Ki67 E), GAP‐43 F), N‐cadherin G), vinculin H), and integrin I) expression of RSCs on the GO/PCL and PCL scaffolds. Three independent replicates were included for each group. Relative mRNA expression is shown compared with GAPDH. *P < 0.05 compared with PCL.

We also comprehensively evaluated the proliferation and attachment of cells on the GO/PCL scaffolds. Ki67 is a nuclear protein that is highly involved in cell proliferation. Ki67 immunofluorescence indicated greater cell proliferation on the GO/PCL scaffolds (Figure 5) than on the PCL nanoscaffolds. qPCR and flow cytometry (FCM) both showed that the Ki67 expression of cells cultured for 4 d on the GO/PCL nanoscaffolds was increased by 1.8‐fold compared with those cultured on the PCL nanoscaffolds (**Figure** **6** and Figure S2, Supporting Information). Western blot also showed higher expression levels of various proteins in cells cultured on the GO/PCL scaffolds than in those cultured on the PCL scaffolds (Figure 6). Some adhesion‐related genes, such as N‐cadherin, vinculin, and integrin, were also analyzed by qPCR. N‐cadherin is a transmembrane protein that forms adherence junctions between cells for interlinking and, thus, plays a notable role in cell adhesion. The expression of N‐cadherin by cells cultured on GO/PCL nanoscaffolds cultured for 4 d was 1.2‐fold greater than that of cells cultured on PCL nanoscaffolds (Figure 6). Vinculin serves as a significant link between the actin cytoskeleton and adhesion molecules. The expression of vinculin by cells cultured on the GO/PCL nanoscaffolds cultured for 4 d was increased by 1.2‐fold compared with that of cells cultured on the PCL nanoscaffolds (Figure 6). Integrin also has important implications for cell‐extracellular matrix (ECM) connections. The expression of integrin by cells cultured on the GO/PCL nanoscaffolds cultured for 4 d was increased by 1.9‐fold compared with that of cells cultured on the PCL nanoscaffolds (Figure 6). These results confirmed the good ability of RSCs to adhere to the GO/PCL nanoscaffolds.

**Figure 7:**
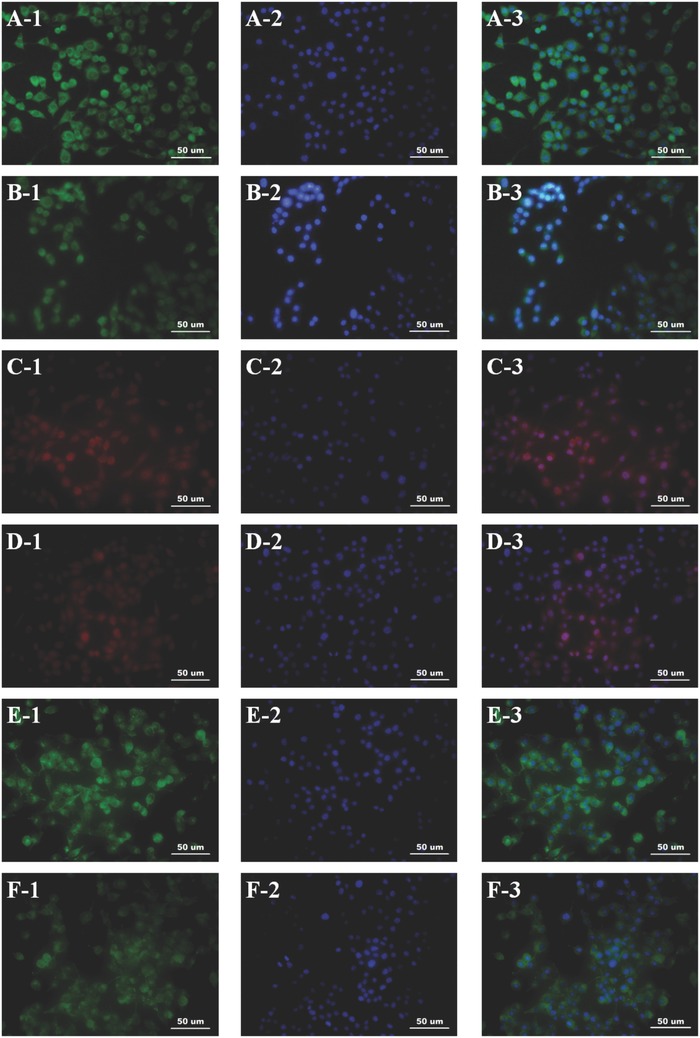
Immunofluorescence staining for S100 A,B), GFAP C,D), and nestin E,F). All samples were washed three times, fixed with 4% paraformaldehyde for 20 min at 25 °C and were blocked with BSA overnight. DAPI staining appears blue. GO/PCL scaffolds (A1–A3, C1–C3, and E1–E3). PCL scaffolds (B1–B3, D1–D3, and F1–F3). The scale bar is 50 µm.

To confirm the impact of the GO/PCL nanoscaffold on RSC neural expression, we assessed the neural specific proteins, glial fibrillary acidic protein (GFAP), β III tubulin (Tuj1), and nestin, as well as the Schwann cell marker S100. GFAP and nestin are both intermediate filament proteins highly expressed by nerve cells. Tuj1 can distinguish neurons from glial cells as a neuron‐specific protein. The immunofluorescence results are displayed in **Figures** 5 and **7**. In addition, we performed qPCR to analyze and quantify the expression of GFAP, Tuj1, nestin, S100, and growth‐associated protein (GAP‐43). GAP‐43 is a growth protein that is highly expressed in nervous tissues and is vital to axonal and synaptic development. The expression levels of GFAP, Tuj1, nestin, GAP‐43, and S100 were increased by 1.1‐fold, 2.4‐fold, 1.8‐fold, 3.5‐fold, and 1.8‐fold respectively, on the GO/PCL nanoscaffolds compared with the PCL nanoscaffolds. For further validation, the relevant Western blot results are also shown (Figure 6). In summary, the GO/PCL scaffolds increased the expression of neuronal markers to a small extent.

**Figure 8:**
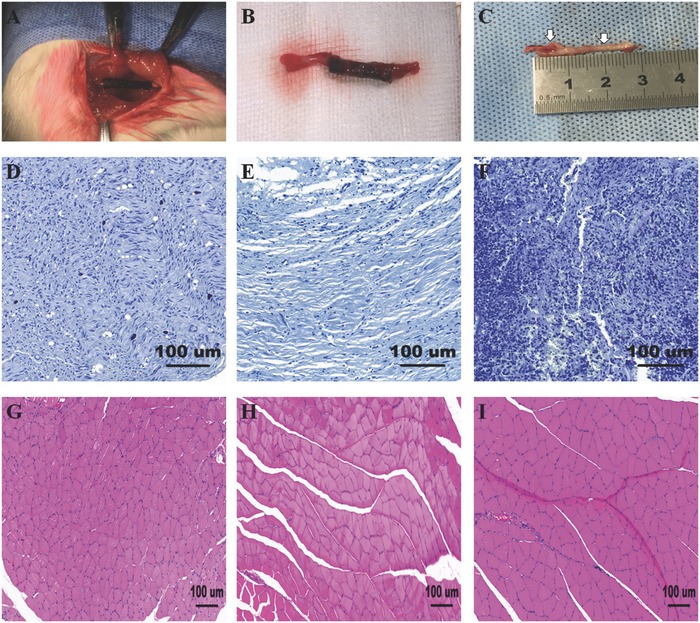
Morphological evaluation of sciatic nerve and muscle regeneration at 18 weeks postoperatively. Optical images of the GO/PCL NGC at implantation A) and at 18 weeks after surgery B), as well as a dissected regenerated nerve section from the in vivo study C). Toluidine blue staining of a GO/PCL conduit D), a PCL conduit E), and an autograft F) at 18 weeks postoperatively. All samples were dissected from 15 mm sections of regenerated nerves. Ultra‐thin 5 µm thick sections were created using a cryostat. The gastrocnemius muscle from the injured side was also collected at 18 weeks postoperatively. The results for the GO/PCL conduits G), PCL conduits H), and autografts I) are displayed. The scale bar is 100 µm.

We further included a long‐term in vivo Sprague Dawley rat model in this study. Rats were randomly divided into three groups a GO/PCL conduit group, a PCL conduit group and an autograft group. All observations and examinations were completed at weeks 6, 12, and 18 postoperatively. No animals suffered an infection through postoperative day 7. No rats showed signs of edema, ulcers or failed surgical wound healing. None of the nerve conduits had degraded by 18 weeks after surgery. The GO/PCL conduit at implantation and the regenerated nerves at 18 weeks postoperatively in rats that received the GO/PCL conduit are displayed in **Figure** **8**.

For the functional and electrophysiological evaluations, we used walking track analysis and electrophysiological assessment methods. At 6 weeks after surgery, the sciatic function index (SFI) of the GO/PCL group (−33.2) was notably higher than that of the PCL group (−39.1, *p* < 0.05) and lower than that of the autograft group (−29.0, *p* < 0.05). Similar results were observed at 12 weeks postoperatively. The SFI of the GO/PCL, PCL and autograft groups was −9.5, 14.4, and 6.5, respectively. At 18 weeks, the SFI of the GO/PCL group was not significantly different from that of the autograft group (*p* > 0.05) (Figure S3, Supporting Information). Significantly less gastrocnemius muscle regeneration was observed in the PCL group than in the GO/PCL and autograft groups (*p* < 0.05). No notable difference was found between the GO/PCL and autograft groups (*p* > 0.05).

The results of the electrophysiological analysis at 6 weeks postoperatively revealed that the nerve conduction velocity (NCV) of the GO/PCL group (13.2 m s^−1^) was notably higher than that of the PCL group (11.2 m s^−1^, *p* < 0.05) and significantly lower than that of the autograft group (17.3 m s^−1^, *p* < 0.05). At 12 weeks after surgery, the NCV was 20.8, 17.2, and 25.3 m s^−1^ in the GO/PCL, PCL, and autograft groups, respectively. At 18 weeks, the NCV of the GO/PCL group (33.4 m s^−1^) was similar to that of the autograft group (37.3 m s^−1^, *p* > 0.05) and superior to that of the PCL group (29.3 m s^−1^, *p* < 0.05). At 6 weeks after surgery, the distal compound motor action potential (DCMAP) of the PCL group (7.1 mV) was significantly lower than that of the GO/PCL group (9.3 mV, *p* < 0.05) and the autograft group (12.3 mV, *p* < 0.05). This trend was also observed at 12 weeks postoperatively. At 18 weeks, the DCMAP of the GO/PCL group (25.1 mV) was not significantly different from that of the autograft group (27.4 mV, *p* > 0.05) (Figure S3, Supporting Information).

**Figure 9:**
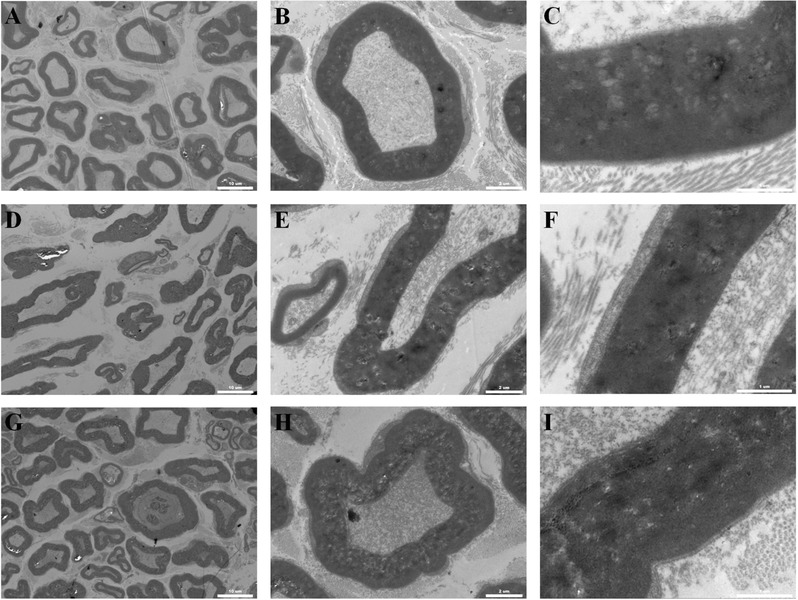
TEM images for transverse sections of regenerated nerves from a GO/PCL conduit A–C), a PCL conduit D–F), and an autograft G–I) at 18 weeks postoperatively. We evaluated cross‐sections from different samples and used uranyl acetate and lead citrate for staining. All specimens were observed by TEM. The scale bars are 10 µm A, D, and G), 2 µm B, E, and H) and 1 µm C, F, and I), respectively.

TEM and toluidine blue staining were performed to validate the morphological improvement in the different groups. The representative sections that were included in the following experiments are displayed in Figure 8C. In general, the total number, area, diameter, and thickness of regenerated nerves and myelinated axons of the GO/PCL group were markedly higher than those of the PCL group (*p* < 0.05) at 6, 12, and 18 weeks after surgery and were similar to those of the autograft group at 18 weeks despite significant differences at 6 and 12 weeks after surgery (**Figures** 8 and **9** and Figures S4 and S5, Supporting Information). Toluidine blue staining also revealed significantly more nerve fibers in the GO/PCL group than in the PCL group at 18 weeks after implantation (*p* < 0.05, Figure 8). TEM further indicated that the thickness of myelinated axons in the GO/PCL group was significantly greater than that of the PCL group at 18 weeks (*p* < 0.05). However, there was no notable difference in the myelinated axon thickness between the GO/PCL and autograft groups at 18 weeks (*p* > 0.05, Figure 9).

The sciatic nerve controls the gastrocnemius muscle. Therefore, the functional regeneration of the sciatic nerve can be reflected by atrophy of the gastrocnemius muscle.^35^ Random fields of view in images of different tissue sections were selected to identify muscle fibers using Image‐Pro Plus software. The percentage of muscle fiber area (Pm) was assessed using the equation Pm = Am/At × 100%, where Am is the mean area of muscle fibers, and At is the total area of the field.^36^ The mean area of muscle fibers was much larger in the autograft and GO/PCL conduit groups than in the PCL group (*p* < 0.05). The mean area of muscle fibers was not significantly different between the GO/PCL and autograft groups (*p* > 0.05, Figure 8 and Figure S4, Supporting Information), indicating that GO/PCL can reverse muscle atrophy to some extent in long‐range defects of the sciatic nerve.

**Figure 10:**
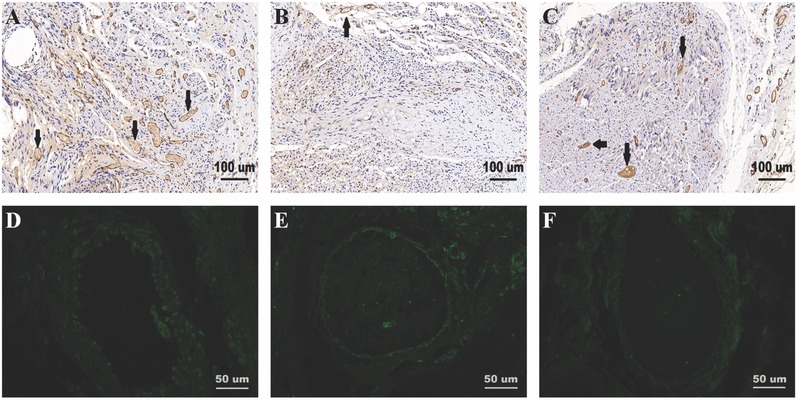
Assessment of angiogenesis in sciatic nerve regeneration at 18 weeks postoperatively. Immunohistochemistry staining for CD31 in regenerated nerve samples from a GO/PCL conduit A), a PCL conduit B), and an autograft C) at 18 weeks after surgery. CD31 is important for endothelial cell intercellular junctions and is extensively involved in angiogenesis. CD31^+^ cells are indicated by arrows in each picture. CD34 is a transmembrane protein that is associated with vascular tissues. Immunofluorescence staining for CD34 in regenerated nerve samples is also shown for a GO/PCL conduit D), a PCL conduit E), and an autograft F) at 18 weeks after surgery. The scale bars are 100 µm A–C) and 50 µm D–F), respectively.

**Figure 11:**
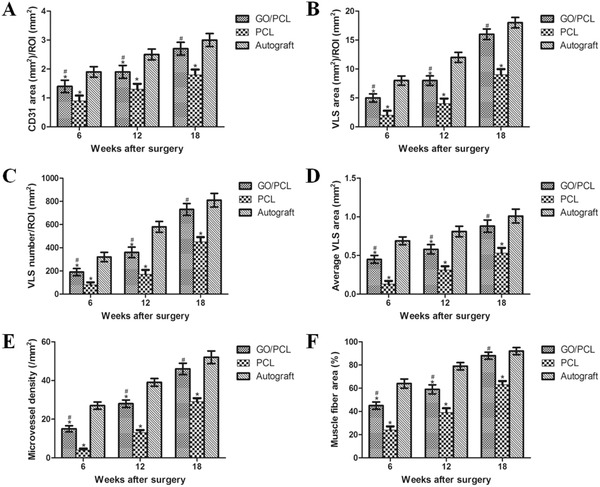
Quantification of CD31^+^‐ and CD34^+^ cells based on various measurements of sciatic nerve samples from the GO/PCL conduit, PCL conduit and autograft groups at 6, 12, and 18 weeks postoperatively. CD31 area (mm^2^)/region of interest (ROI) (mm^2^) A). VLS area (mm^2^)/ROI (mm^2^) B). VLS number/ROI (mm^2^) C). Average VLS area (mm^2^) D). Quantification of CD34^+^ region via MVD (per mm^2^) E). Quantification of muscle fiber area (%) (F). *P < 0.05 compared with autograft; ^#^ *P* < 0.05 compared with PCL conduit.

Microvessels are vital to Schwann cell migration and peripheral nerve regeneration. To verify the influence of the different scaffolds on angiogenesis in the process of nerve regeneration, various markers were used including CD34 and CD31. CD34 is a hematopoietic transmembrane protein that is closely associated with vascular‐associated tissue. CD31 is also known as platelet endothelial cell adhesion molecule (PECAM‐1) which actively participates in endothelial cell junction formation and angiogenesis. The microvessel density (MVD) was assessed by immunostaining for CD34, as shown in **Figure** **10** and Figure S6 in the Supporting Information. The MVD was significantly lower in the PCL group than in the GO/PCL and autograft groups (*p* < 0.05), and while it was slightly higher in the autograft group than in the GO/PCL group, this difference was not significant (*p* > 0.05, **Figure** **11**).

CD31 staining was performed via immunohistochemistry assays as previously described.^37^ The CD31^+^ area, vessel‐like structure (VLS) area and density ((VLS area+ CD31^+^ area)/total scaffold area) were better in the autograft and GO/PCL groups than in the PCL group. There were no significant differences in these metrics between the autograft and GO/PCL groups (*p* > 0.05, Figures 10 and 11 and Figure S6, Supporting Information).

**Figure 12:**
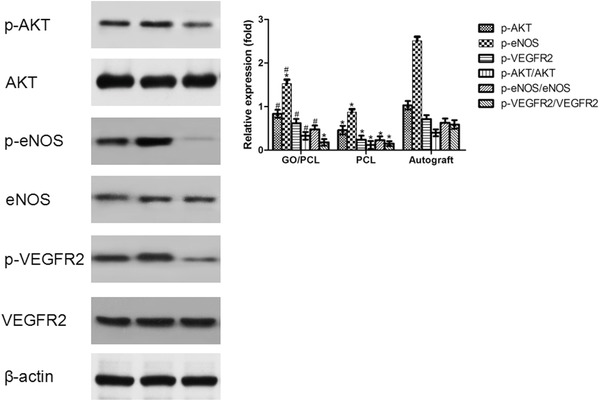
Western blot results for AKT, p‐AKT, eNOS, p‐eNOS, VEGFR, and p‐VEGFR expression in regenerated nerves from the GO/PCL conduit, PCL conduit and autograft groups at 18 weeks after surgery. The relative expression shown was normalized to that of β‐actin. From left to right: GO/PCL conduit, autograft and PCL conduit. *P < 0.05 compared with autograft; ^#^ *P* < 0.05 compared with PCL conduit.

After demonstrating the angiogenic capability of the GO/PCL NGC in sciatic nerve regeneration, we further considered the potential mechanism behind this important phenomenon. We purified proteins from the regenerated sciatic nerves of the Sprague‐Dawley rats for western blot at 18 weeks postoperatively in the three experimental groups. We evaluated the expression of AKT, p‐AKT, endothelial nitric oxide synthase (eNOS), p‐eNOS, vascular endothelial growth factor receptor (VEGFR) 2, and p‐VEGFR2 with β‐actin as a loading control. The WB pictures were displayed in **Figure** **12**. From the pictures, there were no obvious differences in AKT expression among the GO/PCL, PCL and autologous groups. p‐AKT expression was significantly lower in the PCL group than in the GO/PCL and autologous groups (*p* < 0.05). Similarly, there were no significant differences in eNOS or VEGFR2 among the three groups (*p* > 0.05). The expression levels of p‐eNOS and p‐VEGFR2 were notably elevated in the GO/PCL and autologous groups compared with the PCL group, reflecting the potential role of GO instead of PCL in regulating the eNOS and VEGF signaling pathway activation. Comparing all the evaluated proteins revealed that GO contributed to a definitive improvement in AKT signaling pathway activation, thus, initiating downstream NO activation as confirmed by the upregulation of p‐eNOS, finally leading to the stimulation of VEGF expression and contributing to angiogenesis in the injured peripheral nerve.

The regeneration process of long‐range nerve defects primarily relies on a physical bridge between two nerve stumps and the chemical guidance of bioactive molecules and proteins.^38^ NGCs have been widely accepted and used as a successful biomaterial in studies of peripheral nerve injury.^39^ Masand fabricated a peptide‐modified nerve conduit with polysialic acid and a human natural killer cell epitope and found that it was highly effective for promoting myelination, axonal growth and motor neuron recovery in a mouse femoral nerve defect model.^40^ Huang used an active silk conduit in rat sciatic nerve repair and discovered that long gaps, including 11 and 13 mm gaps, could be successfully repaired by 12 weeks after injury.^41^ Wu fabricated a bioactive polyurethane nanoscaffold and discovered that it upregulated neurotrophin expression by activating voltage‐gated calcium channels to improve peripheral nerve regrowth.^42^ Wang fabricated nerve scaffolds with different stiffnesses and evaluated their roles in peripheral nerve regeneration. In their research, higher PCL concentrations in the poly(propylene fumarate)‐co‐PCL scaffold better improved peripheral nerve function and structural repair.^43^ However, to achieve fully functional and structural recovery, an advanced bioactive scaffold is needed to provide an ideal environment for nerve tissue regrowth. Conductive nanoscaffolds have the advantage of promoting electrical signal transduction in cell‐cell junctions.^44^ GO is a representative new conductive material with the ability to promote cell attachment,^45^ proliferation,^46^ and more importantly, angiogenesis.^47^ The integration molding method enables GO and PCL to be mixed to fabricate an integrated scalable nanoscaffold, which imparts great convenience and efficiency.^48^ In addition, integrated fabrication can successfully solve many problems of traditional electrospinning method, including the uneven distribution of GO nanoparticles, weak rigidity and elasticity, and imbalanced quality control. The rough and wrinkled surface allows GO to mimic the natural state of the ECM. Thus, RSCs can adhere, proliferate and migrate on GO/PCL nanoscaffolds. In our study, vinculin, N‐cadherin and integrin were used to evaluate cell adhesion on the GO/PCL nanoscaffolds. The results showed that the GO/PCL nanoscaffolds contributed to favorable RSC attachment due to excellent simulation of the cell‐matrix interface. Apart from the cell morphology on the scaffolds, we also observed cross‐sectional images of GO/PCL sheets. Very few cells grew within the scaffolds and exhibited disorganized shapes (Figure S7, Supporting Information). This revalidated that RSCs exhibited firm attachment to the GO/PCL nanoscaffold.

The multiple pores of the GO/PCL nanoscaffolds permit the free exchange of nutrients, such as necessary proteins, oxygen and water, thus facilitating the delivery of energy for nerve regeneration. It was previously reported that porous nanoscaffolds could mediate cellular functions in vitro, such as proliferation and migration.^49^ Ki67, which is an important indicator of the proliferative state of cells, was used in our study to show that the GO/PCL scaffolds promoted cell proliferation. The enhanced proliferation was further demonstrated by western blot, qPCR and immunofluorescence assays.

GO has multiple functional groups that can reduce its cytotoxicity to different cells and tissues. However, the functional groups also sacrifice some of the mechanical and electrical properties of GO, thus diminishing the ability of the material to maintain neural cells. In this study, Tuj1, GFAP, GAP‐43 and nestin were used to evaluate the influence of the GO/PCL scaffolds on RSCs. The results revealed slightly elevated levels in these proteins in cells cultured on the GO/PCL nanoscaffolds compared with those cultured on PCL nanoscaffolds, which might result from relatively low concentration of GO in the scaffold. At the meantime, we found better RSC performance of proliferation, angiogenesis and neural expression in GO/PCL and PCL scaffolds compared with other traditional substrate materials in nerve regeneration, such as PLGA (poly(lactic‐co‐glycolic acid)) and collagen (Figures S8 and S9, Supporting Information). This revalidated that PCL was a common and advantageous system and the addition of GO further improve the excellent performance in nerve tissue engineering.

This is the first study to evaluate the ability of GO to promote long‐term peripheral nerve regeneration in vivo. A rat model of peripheral nerve injury was established by creating a 15 mm defect in the sciatic nerve to determine whether GO could fully repair the injury within 18 weeks. The functional and electrophysiological evaluation confirmed that the GO/PCL scaffolds better promoted axonal regeneration than did the PCL scaffolds. In the histological examination, the total number, area, diameter and thickness of regenerated nerves and myelinated axons were higher in the GO/PCL group than in the PCL group. Moreover, the gastrocnemius muscle, which is manipulated by the sciatic nerve, also exhibited less atrophy in the GO/PCL conduit group than in the PCL conduit group, indicating the role played by GO in neural repair. We also analyzed high GO concentration in PCL scaffold for long‐term peripheral nerve regeneration. The morphological results showed significant toxicity of 2% and 4% GO in PCL led to poor nerve regrowth (Figure S10, Supporting Information).

Angiogenesis is a necessary process during which new blood vessels appear and grow in the original vessel channel. Angiogenesis plays a vital role in many important physiological mechanisms, such as cardiovascular diseases, wound healing and cancer.^50^, ^51^, ^52^ In peripheral nerve injury, angiogenesis also serves as an important agent for nerve repair as it can offer a physical link between nerve stumps, provide injured nerves with fresh nutrients and help remove wastes produced in the physiological process, during which ROS are activated as a result of regional inflammation.^53^ Some studies have reported that low ROS concentrations may be pro‐angiogenic. However, high ROS concentrations may be anti‐angiogenic.^54^ Furthermore, it is difficult to manipulate ROS. In addition to ROS, NO, regulates angiogenesis in various cardiovascular diseases,^55^ and NOS facilitates NO release from L‐arginine. Of the three isoforms of NOS, eNOS produces NO in blood vessels and participates in mediating vascular function. In addition, the activity of PI3K and AKT is correlated and these molecules further activate eNOS in the mediation of angiogenesis, which has become widely studied by many researchers.^56^, ^57^, ^58^

In this study, we focused on the degree of angiogenesis and its underlying mechanism in a rat sciatic nerve injury model. After 18 weeks, regenerated sciatic nerves from the three experimental groups were dissected for evaluating angiogenesis. CD31 and CD34 were selected as immunochemistry markers. CD31, also termed PECAM‐1, was discovered on the superficial layer of platelets, neutrophils, and some T cells and has been confirmed to be present in endothelial cell intercellular junctions.^59^ CD34, which belongs to a family of a single‐pass transmembrane proteins, was found in association with early hematopoietic and vascular‐associated tissue.^60^ Different MVDs were found in the three groups by CD34 staining. Furthermore, CD31 contributes to the strong angiogenic power of GO in the process of sciatic nerve regeneration. By measuring the VLS area and density, the degree of angiogenesis of the regenerated nerves in the GO/PCL conduits was further quantified.

We then sought to identify the potential mechanism behind the pro‐angiogenic characteristic of the GO/PCL nanoscaffolds. The AKT protein expression level did not significantly differ among the GO/PCL, PCL and autograft groups. However, the p‐AKT expression level was markedly higher in the GO/PCL group than in the PCL group, indicating initiation of the AKT signaling pathway by GO. Similarly, p‐eNOS was highly upregulated in the GO/PCL group compared with the PCL group, while eNOS was not. This finding is consistent with previous publications showing that eNOS is a downstream protein regulated by the AKT signaling cascade.^61^, ^62^ VEGF is widely considered a major element of angiogenesis initiation. The binding of VEGF with VEGF receptors (e.g., VEGFR2) induces endothelial cell proliferation, adhesion and migration and further mediates activation of the angiogenic process downstream of NO to improve vessel permeability. We also confirmed p‐VEGFR2 activation in vivo by western blot. Thus, AKT‐eNOS‐VEGF signaling was confirmed to be involved in the angiogenic ability of GO in the process of peripheral nerve regeneration. Several other molecules stimulate angiogenesis, including bFGF, Ang1, Ang2, and ephrin. The mechanism discussed above is only one possible signaling cascade. In future work, we will focus on determining other leading factors that may govern the healing and regrowth of vessels, which has major implications for peripheral nerve regeneration.

Material fate is very vital to a successful nerve scaffold and functional nerve regeneration. We also evaluated the gradual GO release and PCL degradation in the long‐term in vivo study. The GO was released along with PCL degradation due to its nanoscale. Nanosized GO hardly diffused to the environment in the long‐term in vivo experiment from PCL scaffold. In this way, we displayed the PCL biodegradation and GO release at different time points in vivo. The details were shown in Table S2 and Figure S11 in the Supporting Information. To correlate GO release profile with tissue reconstruction efficiency, we performed the graph concerning the correlation of GO release and peripheral nerve and vascular regeneration at 6, 12, and 18 weeks after surgery. The increase of GO release is accompanied by increased nerve morphological recovery and angiogenic restoration (Figure S12, Supporting Information). This validates our previous results and statement that GO/PCL scaffold can improve long‐range peripheral nerve defect effectively and this phenomenon is closely related with GO release to the surrounding environment.

In conclusion, we successfully created GO/PCL NGCs using the integration molding method. Our NGC combines an excellent conductive material GO and a biodegradable stiff material PCL fabricated by 3D integration molding method. Our scaffold design takes biochemical cues, electrical cues and topographical cues into account, which are major factors to make a successful nerve guidance conduit in the peripheral nerve tissue engineering.^63^ Our in vitro study indicated that GO promoted cell attachment, cell proliferation and neural property maintenance. The in vivo study confirmed that the GO/PCL NGC and autologous nerves promoted healing to a similar extent in a 15 mm sciatic nerve defect model. Therefore, GO/PCL NGCs address current drawbacks associated with nerve guidance and neurite regeneration and have huge potential for use in peripheral nerve regeneration.

## 3 Conclusion

In this study, the effects of GO/PCL nanoscaffolds in nerve repair were evaluated both in vitro and in vivo, and the results showed excellent functional and morphological recovery equivalent to those of autografts. We focused on the pro‐angiogenic characteristic of GO and the potential mechanism behind this key phenomenon. The nanoscaffolds directly contributed to successful functional restoration in a long nerve defect model. We plan to continue studying the complex interplay between GO nanomaterials and nerve regeneration.

## 4 Experimental Section

*Integration Molding Method for Producing GO‐Coated PCL Nanofiber Scaffolds and Conduit*: GO and PCL were purchased from Sigma Aldrich. The GO nanoparticles were mixed with PCL in a uniform solution and sonicated for 5 min. The injectable suspension was then contained in a previously designed mold and further prepared by a jet spraying process. Using a nozzle and compressed air, the solution was sprayed from a collection container. The dichloromethane evaporated with the formation of the GO/PCL membrane. The integration molding method was used to create a multi‐layered GO/PCL conduit. Finally, a 3D printer was used to create various evenly distributed pores in the surface of the GO/PCL conduit.

*Characterization of the GO/PCL Conduit*: The GO/PCL nanoscaffold was examined by SEM to evaluate the surface structure and by TEM to examine the GO nanoparticle distribution. Samples were coated with gold prior to observation. A Sirion 200/IAC SEM system was used for observation at an accelerating voltage of 5 kV. Images were captured at 100×, 2000×, and 5000× magnification. A JEOL JEM‐2010 (HT) electron microscope was used for TEM. The images were selected at different magnifications and random fields of view for the final assessment. The molecular orientation of two scaffolds was evaluated using transmission FTIR spectrometer (Nexus670, ThermoNicolet). In addition, the surface elastic modulus and elongation at break were measured by nanoindentation (Nano Indenter G200, Agilent, USA). At least six indentations were recorded for the final statistical evaluation. In addition, the conductive capability was measured via a four‐point probe method using a Hall Effect Test System (DX3000, Dexing Magnet Tech, China).

*RSC Culture and Proliferation Assay*: RSCs were purchased from the cell bank of the Chinese Academy of Sciences (Shanghai, China). RSCs were cultured in high‐glucose Dulbecco's modified Eagle's medium supplemented with 10% fetal bovine serum (Gibco, USA) and 1% penicillin/streptomycin solution (Gibco, USA). Cells were incubated in a humidified atmosphere at 37 °C and 5% CO_2_. The GO/PCL nanoscaffolds were sterilized by a 4 h immersion in ethyl alcohol and overnight exposure to ultraviolet light. Cells were seeded on the nanoscaffolds at a density of 2 × 10^4^ cm^−2^. CCK8 was used to assess cell proliferation. To determine the optimal GO percentage in the PCL scaffolds, the cells were cultured on 0.5% GO/PCL, 1% GO/PCL, 2% GO/PCL, 4% GO/PCL, and PCL scaffolds in 24‐well plates for 1, 3, 5, and 7 d. The medium was replaced every 2 d. Twenty microliters of CCK‐8 solution was added to 200 µL of medium in each well, and cells were further cultured in a 5% CO_2_ incubator for 4 h. Then, 100 µL of medium from each well was transferred to a new 96‐well plate and the absorbance at a wavelength of 450 nm was determined using a multifunctional microplate reader (Thermo 3001, Thermo Fischer Scientific, USA). TCP was used as a control. Three independent samples were evaluated with RSCs for each nanoscaffold.

*Cell Viability Assay*: RSCs were cultured on 1% GO/PCL scaffolds, PCL scaffolds and TCP. After 24 h of culture, cells on the different nanoscaffolds were washed gently with phosphate‐buffered saline (PBS). Then, a LIVE/DEAD cell staining kit (Invitrogen) was used to quantify cell viability according to standard protocols. Finally, all samples were observed by an inverted phase contrast microscope.

*Cell Morphology*: RSCs were cultured on GO/PCL and PCL nanoscaffolds for 4 d. The medium was replaced every 2 d. The morphology of the RSCs on the different scaffolds was observed by SEM (Hitachi). First, the medium was removed from the cell culture and replaced with fresh Dulbecco's PBS (Gibco, USA). Cells on the scaffolds were then fixed with 2.5% glutaraldehyde for 12 h at 4 °C. After the fixation solution was removed, 1% osmium acid was added, and the cells were cultured for 2 h at 4 °C. The samples were dehydrated by a graded ethanol series (30%, 50%, 70%, 80%, 90%, 95%, 100%) twice for 20 min each. Lyophilization and gold coating were employed for the final analysis. The samples were observed by SEM to evaluate cell morphology and attachment on the scaffolds.

*Immunofluorescence*: After 4 d of cell culture, the cells on the scaffolds were gently washed with PBS three times, fixed in 4% paraformaldehyde for 30 min, and immersed in 0.1% Triton X‐100 (Sigma) for 5 min. The samples were then blocked with 5% bovine serum albumin (BSA) and incubated with primary antibodies overnight at 4 °C followed by 2 h of incubation with secondary antibodies at room temperature. Finally, 4,6‐diamidino‐2 phenylindole (DAPI) (1:500, Gibco, USA) was used to stain the nuclei. The primary antibodies were anti‐nestin (1:100, Abcam, USA), anti‐Tuj1 (1:500, Abcam, USA), anti‐GFAP (1:1000, Abcam, USA), anti‐Ki67 (1:250, Abcam, USA), and anti‐S100 β (1:100, Abcam, USA). The secondary antibody was Alexa Fluor 488‐conjugated mouse anti‐rabbit IgG (1:200, Gibco USA). For the cell attachment analysis, F‐actin was stained after the cells were cultured for 4 d on different scaffolds (GO/PCL and PCL). The procedures were similar to those described above. Phalloidin conjugated to Alexa Fluor 488 (1:200, Abcam, USA) was used to stain actin filaments. All samples were observed by inverted phase contrast microscopy.

*WB, FCM, and RT‐qPCR*: Western blot was conducted as follows. Cells were lysed in RIPA lysis buffer to collect total proteins. SDS‐PAGE was performed, and the samples were transferred onto PVDF membranes. The samples were then incubated at 4 °C overnight with the following primary antibodies: anti‐GAP‐43 (1:10000, Abcam USA), anti‐GFAP (1:10000, Abcam, USA), anti‐Ki67 (1:5000, Abcam, USA), anti‐Tuj1 (1:1000, Abcam, USA), anti‐nestin (1:2000, Abcam, USA), anti‐S100 β (1:5000, Abcam, USA), anti‐N‐cadherin (1:5000, Abcam, USA), anti‐vinculin (1:10000, Abcam, USA), and anti‐integrin α5 (1:1000, Gibco, USA). The Ki67 expression was also evaluated using FCM.

Total RNA was extracted from the cells using TRIzol reagent (Gibco, USA) according to the manufacturer's protocol. RNA was reverse‐transcribed into cDNA with PrimeScriptTM (Takara). According to the manufacturer's instructions, samples were run on a real‐time PCR biosystem. The primer sequences were N‐cadherin (Forward (5′‐3′) CAGGGCCCTTTGCATTTGAC), integrin (Forward (5′‐3′) TGTCCTACTGGTCCCGACAT), vinculin (Forward (5′‐3′) TGGTCTAGCAAGGGCAATGAC), Ki67 (Forward (5′‐3′) ACAGGGCTTAGGAAACAGTCC), nestin (Forward (5′‐3′) GGGGGTAGGAGATGCCTTTG), GFAP (Forward (5′‐3′) TGCATGTACGGAGTATCGCC), GAP‐43 (Forward (5′‐3′) ACCTAAGGAAAGTGCCCGAC), Tuj1 (Forward (5′‐3′) AGCTCACCCAGCAGATGTTC), S100 (Forward(5′‐3′), CGATGCCCCGGAAAGTTAGA), and GAPDH (Forward(5′‐3′), GGCAAGTTCAACGGCACAGT).

*Animal Surgery*: 45 male Sprague Dawley rats (weighing 150–200 mg) were housed in a specific pathogen‐free atmosphere. The animals were randomly divided into three experimental groups: a GO/PCL group (15 rats), a PCL group (15 rats), and an autograft group (15 rats). Animals were anesthetized via an intraperitoneal injection of 40 mg kg^−1^ sodium pentobarbital. Under sterile conditions, a small incision was created in the right leg of the rat to expose the sciatic nerve located 4 mm below the skin. The surrounding muscles were detached with blunt dissection. Then, a 15 mm defect was created in the right thigh of each rat for the different implants. A GO/PCL conduit, a PCL conduit or an autologous nerve was sutured to the proximal and distal ends of the injured nerve with 6‐0 nylon sutures. The muscle soft tissue and skin were sutured accordingly with 3‐0 nylon sutures. Then, 800 000 units of penicillin were administered to each rat immediately after surgery to prevent infection. Subsequent postoperative observations were made at week 6, 12, and 18. In this study, all animal care and use were performed according to the guidelines approved by the Institutional Animal Care and Use Committee of Shanghai Jiao Tong University (SJTU, No. A2017072).

*Functional Analysis and Electrophysiological Assessment*: Infection, edema, and surgical wound healing were assessed in each group. Walking track analysis was used to evaluate nerve regeneration based on the following formula: SFI = (−38.3 × (EPL − NPL)/NPL) + (109.5 × (ETS − NTS)/NTS) + (13.3 × (EIT − NIT)/NIT) − 8.8. The foot print length is the distance between the heel and the top of the third toe, and toe spread is the distance from the first to the fifth toe, and intermediary toe spread is the distance between the second and fourth toes. These metrics were determined for the foot on the unoperated side (normal foot print length, NPL; normal toe spread, NTS; normal intermediary toe spread, NIT), and evaluations of the experimental foot print length (EPL), experimental foot toe spread (ETS), and experimental foot intermediary toe spread (EIT) were made for the foot of the operated side. The SFI ranges from −100 to 0, with −100 corresponding to complete nerve dysfunction and 0 representing good repair.

Electrophysiological assessments of the NCV and DCMAP, were conducted immediately after the walking track analysis at 6, 12, and 18 weeks postoperatively. Before starting the electrophysiological recordings, conduits that were not degraded were removed from the regenerated nerves under anesthesia. A monopolar recording device was used to record the NCV and DCMAP with digital electromyographs of the gastrocnemius muscle for all groups.

*Histological Analysis*: Immediately after the electrophysiological evaluations, 15 mm sections of the right regenerated nerves of the experimental rats were dissected. The NGC was carefully removed with the regenerated sciatic nerve remaining inside. Thereafter, the conduit was opened with small scissors to expose the nerve. Nerves were cut into ultra‐thin 5 µm thick sections for further histological evaluation. Both 1% toluidine blue staining and TEM were performed. The regenerated nerve number and thickness, the diameters of myelinated fibers, and the thickness of the myelin sheath were calculated using Image‐Pro Plus, as described in the previous studies. The gastrocnemius muscles of the experimental leg was removed and stained with hematoxylin and eosin. Random fields of view were selected in the images to evaluate the muscle fibers using Image‐Pro Plus, and the muscle was assessed by the percentage of muscle fiber area (Pm) according to the equation: Pm = Am/At × 100%, where Am is the area of muscle fibers, and At is the total area of the field.

*Assessment of Angiogenesis*: At 6, 12, and 18 weeks postoperatively, the middle sections of the regenerated sciatic nerves were dissected, and the transverse layer was used for CD34 immunofluorescence assays. In short, tissues were prepared on a thin slide. The primary antibody was rabbit anti‐CD34 antibody (1:100, Abcam, USA), and FITC conjugated goat anti‐rabbit antibody (1:200, Abcam, USA) was used as a secondary antibody. Quantification was performed by counting the number of microvessels in randomly selected views to evaluate the MVD.

The CD31 staining procedure was as follows. In brief, rehydration of the sections in paraffin by a graded ethanol series was followed by antigen retrieval via heating in sodium citrate. The samples were then incubated in the primary rabbit anti‐human CD31 antibody (1:150, Abcam, USA) in 2% NDS at 4 °C overnight. Then, the slides were incubated with the secondary biotin‐conjugated donkey anti‐rabbit antibody (1:500, Abcam, USA) for 1 h at room temperature. Then, the sections were dehydrated and mounted. Quantification of the pro‐angiogenic characteristic of the GO/PCL scaffolds was based on the VLS area, VLS density, and average VLS area as percentages.

*Western Blot Analysis*: Regenerated nerve tissues were lysed in NP40 lysis buffer supplemented with 1 × 10^−3^ m phenylmethyl sulfonyl fluoride and 1 × 10^−3^ m protease inhibitor cocktail. Samples were subjected to SDS‐PAGE and analyzed by Western blot using specific antibodies for VEGFR2 (1:2000, Abcam, USA), p‐VEGFR2 (1:1000, Abcam, USA), eNOS (1:1500, Abcam, USA), p‐eNOS (Ser1177) (1:1000, Abcam, USA), AKT (1:1500, Abcam, USA), p‐AKT (Ser474) (1:1000, Abcam, USA) and β‐actin (as a loading control). The test was repeated three times, Images were scanned and band density was analyzed using Image‐Pro Plus software, version 6.0. The uncut blots are displayed in Figure S13 in the Supporting Information.

*Material Degradation Measurement*: The nerves were dissected and the remaining GO/PCL nerve conduits were kept at 6, 12, and 18 weeks postoperatively. The weight of the conduits from 6, 12, and 18 weeks after surgery was subtracted from the original GO/PCL conduit to know the total amount of GO release and PCL degradation. In order to quantitatively show the GO release, the 6‐week, 12‐week, 18‐week, and preoperative GO/PCL nerve conduits in the dichloromethane solution (the same volume for each conduit) were dissolved separately. Then, the mixed solution received sonication. Afterward, the GO was extracted by centrifugation. GO sediment weight was measured by vacuum drying it for 24 h. The GO release and PCL degradation amount could be calculated according to the following formula: accumulation GO release amount (mg) = original GO weight (mg) − GO sediment amount (mg); PCL degradation amount (mg) = original conduit weight (mg) − remaining conduit weight (mg) − GO release amount (mg).

*Statistical Analysis*: All tests were repeated three times, and the results are displayed as the mean ± standard deviation. Unpaired Student's *t*‐tests were used for statistical analyses. A *p* value of 0.05 was considered significant.

## Conflict of Interest

The authors declare no conflict of interest.

## Supporting information

Supplementary

Supplementary

Supplementary

Supplementary

Supplementary

Supplementary

Supplementary

Supplementary

Supplementary

Supplementary

Supplementary

Supplementary

Supplementary
