# Supplementary material for: 3D Fabrication with Integration Molding of a Graphene Oxide/Polycaprolactone Nanoscaffold for Neurite Regeneration and Angiogenesis
Source: Adv Sci (Weinh). 2018 Jan 26;5(4):1700499. doi: 10.1002/advs.201700499 (PMC5908351; doi:10.1002/advs.201700499)
Supplement: Supplementary file 1 — Supplementary [file ADVS-5-1700499-s002.pdf]

Copyright WILEY-VCH Verlag GmbH & Co. KGaA, 69469 Weinheim, Germany, 2018.

ADVANCED SCIENCE
Open Access

Supporting Information

for *Adv. Sci.,* DOI: 10.1002/advs.201700499

3D Fabrication with Integration Molding of a Graphene Oxide/Polycaprolactone Nanoscaffold for Neurite Regeneration and Angiogenesis

Yun Qian, Jialin Song, Xiaotian Zhao, Wei Chen, Yuanming Ouyang, Weien Yuan,* and Cunyi Fan**

Three-dimensional printing with integration molding of graphene oxide/ polycaprolactone nanoscaffold for neurite regeneration and angiogenesis

Yun Qian[1,2], Jialin Song[1], Xiaotian Zhao[3], Wei Chen[1], Yuanming Ouyang[1,2*], Weien Yuan[3*], Cunyi Fan[1*]

[1]Shanghai Jiao Tong University Affiliated Sixth People's Hospital, 600 Yishan Road, Shanghai 200233, China
[2]Shanghai Sixth People's Hospital East Campus, Shanghai University of Medicine and Health, Shanghai 201306, China
[3]School of Pharmacy, Shanghai Jiao Tong University, No.800 Dongchuan Road, Shanghai 200240, China

*corresponding to:
Weien Yuan
yuanweien@sjtu.edu.cn or yuanweien@126.com
Yuanming Ouyang
ouyangyuanming@163.com
Cunyi Fan
cyfan@sjtu.edu.cn

Supporting Information

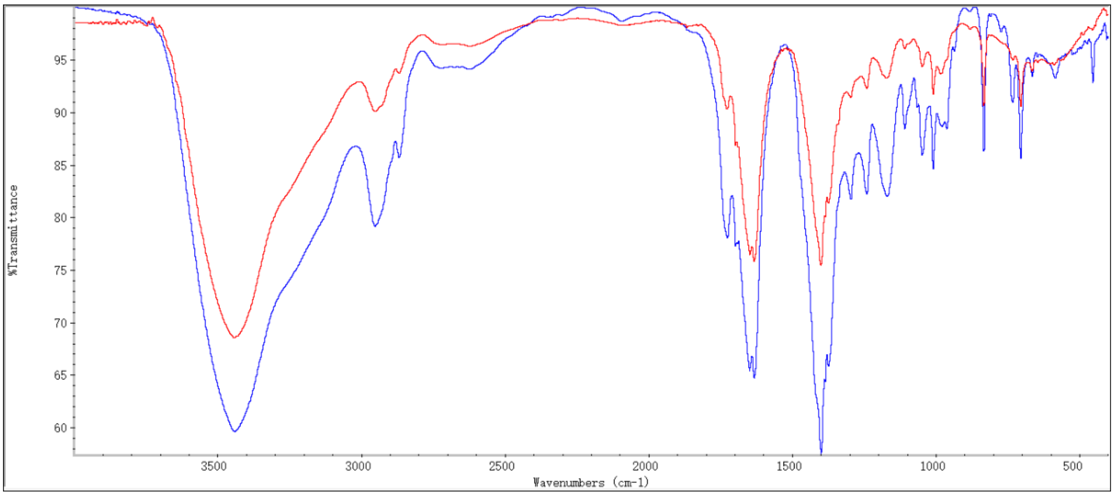

Figure S1. Fourier transform infrared spectra of GO/PCL (blue) and PCL (red) scaffolds.

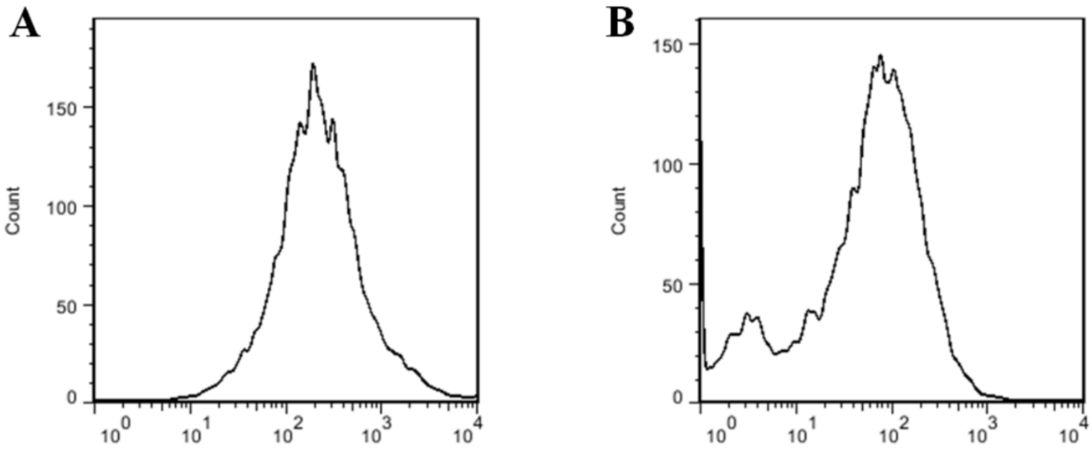

Figure S2. FCM of cell proliferation from GO/PCL (A) and PCL (B) scaffolds.

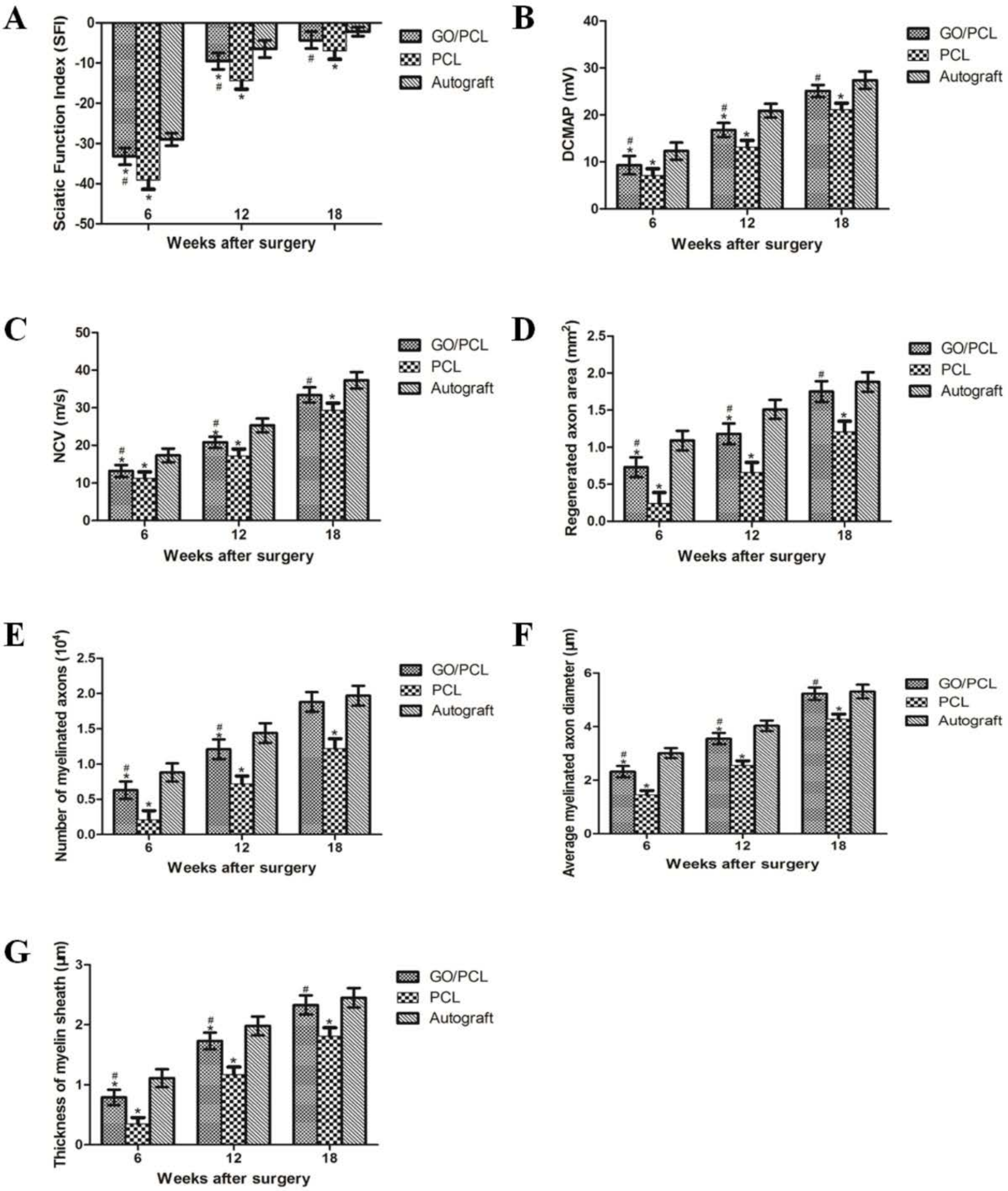

Figure S3. Functional, electrophysiological and morphological evaluations of sciatic nerve regeneration in the GO/PCL conduit, PCL conduit and autograft groups at 6, 12 and 18 weeks postoperatively. The SFI (A) was adopted to measure sciatic nerve functional recovery. The electrophysiological evaluation included the DCMAP (B) and the NCV (C). Major factors of morphological regeneration in the three groups. Regenerated axon area (mm^2) (D). Number of myelinated axons (10^4) (E). Average myelinated axon diameter (μm) (F). Thickness of the myelin sheath (μm) (G).

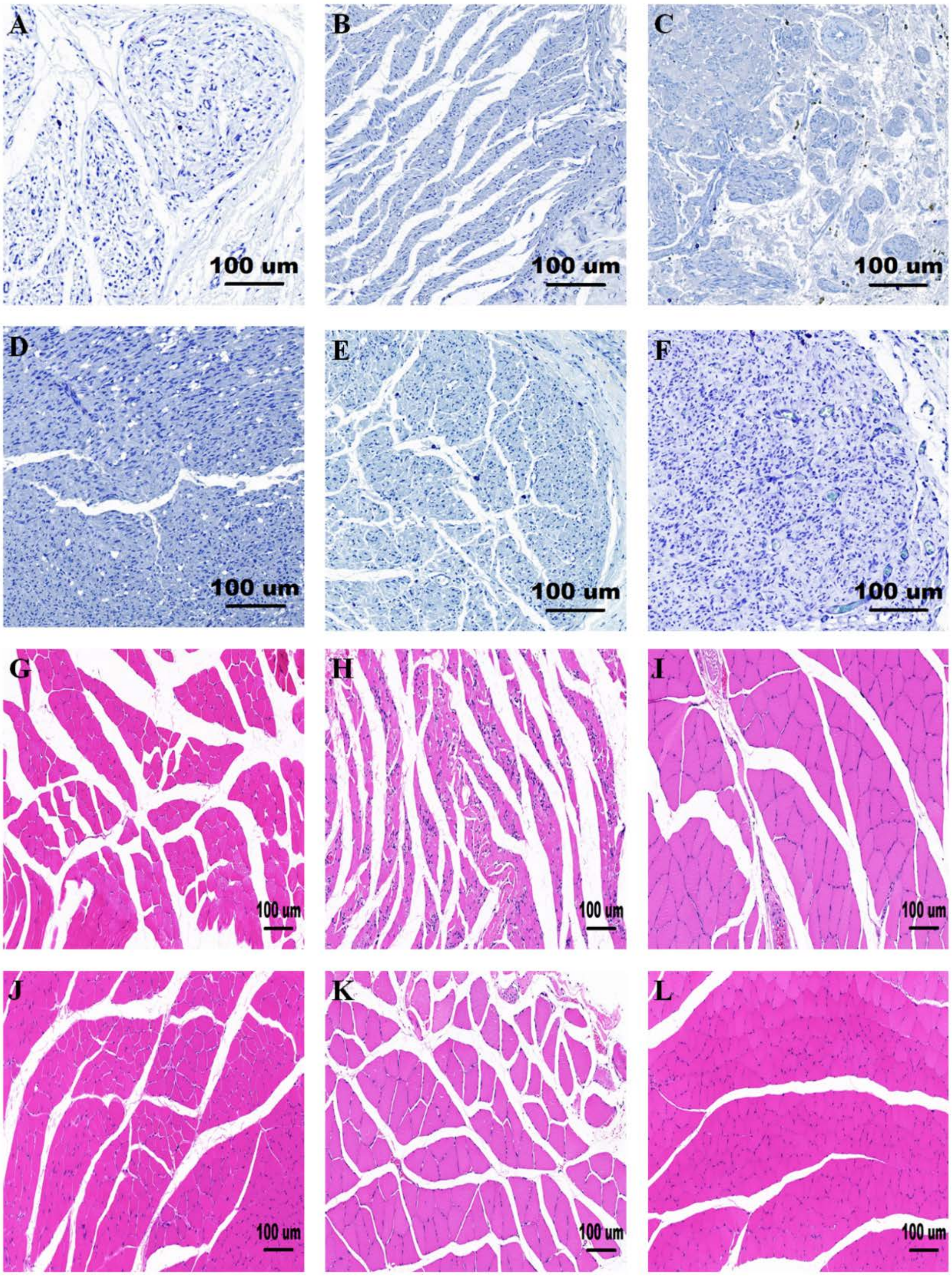

Figure S4. Morphological evaluation of sciatic nerve regeneration at 6 (A, B and C) and 12 weeks (D, E and F) postoperatively. Toluidine blue staining of nerve samples from the GO/PCL conduit (A and D), PCL conduit (B and E) and autograft (C and F) groups. The gastrocnemius muscle from the injured side was also collected at 6 (G, H and I) and 12 weeks (J, K and L) postoperatively. The results of the GO/PCL conduit (G and J), PCL conduit (H and K) and autograft (I and L) groups are displayed. The scale bar is 100 μm.

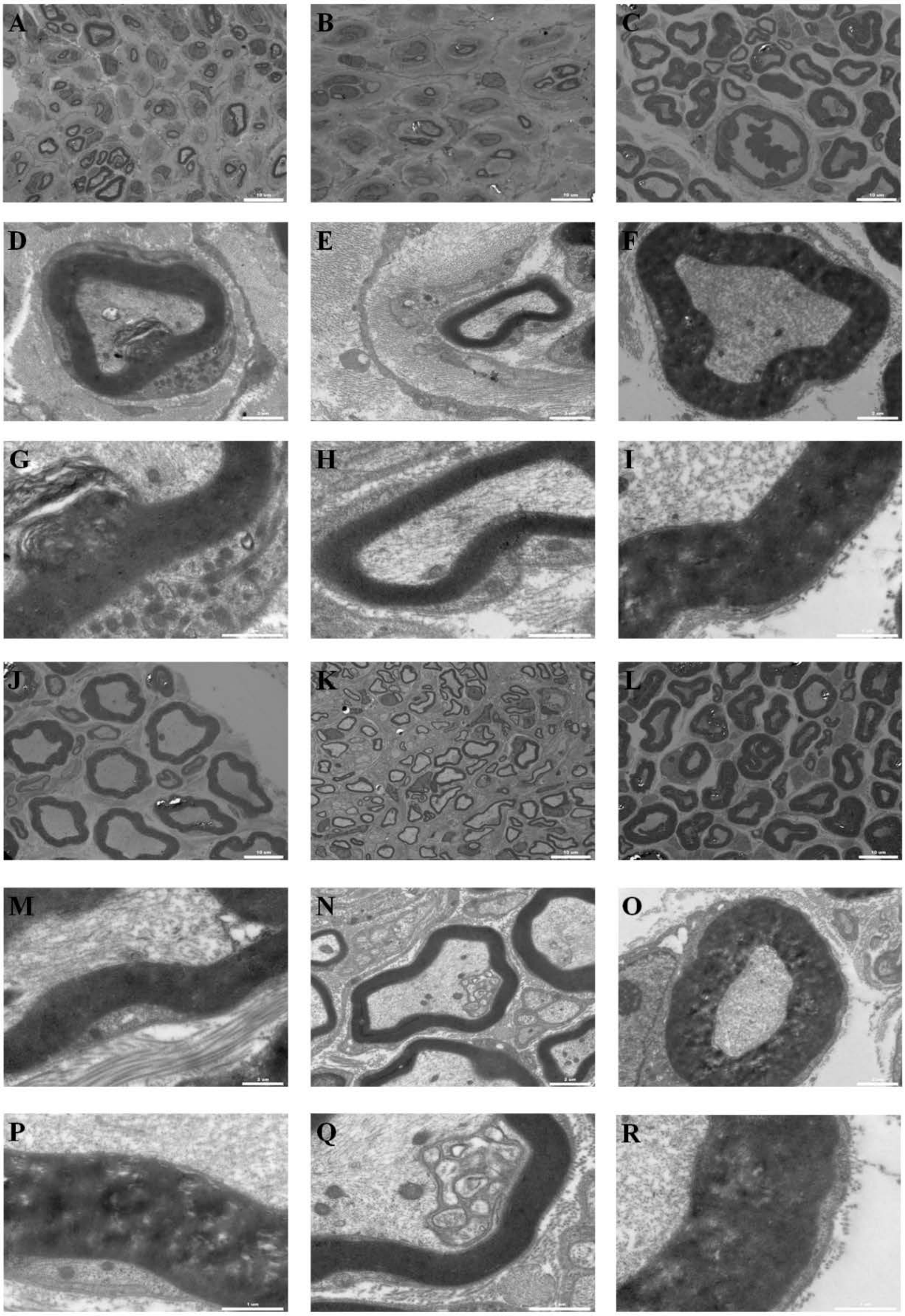

Figure S5. TEM images of transverse sections of regenerated nerves at 6 (A-I) and 12 weeks (J-R) postoperatively from the GO/PCL conduit (A, D, G, J, M and P), PCL conduit (B, E, H, K, N and Q) and autograft (C, F, I, L, O and R) groups. The scale bars are 10 μm (A, B, C, J, K and L), 2 μm (D, E, F, M, N and O) and 1 μm (G, H, I, P, Q and R), respectively.

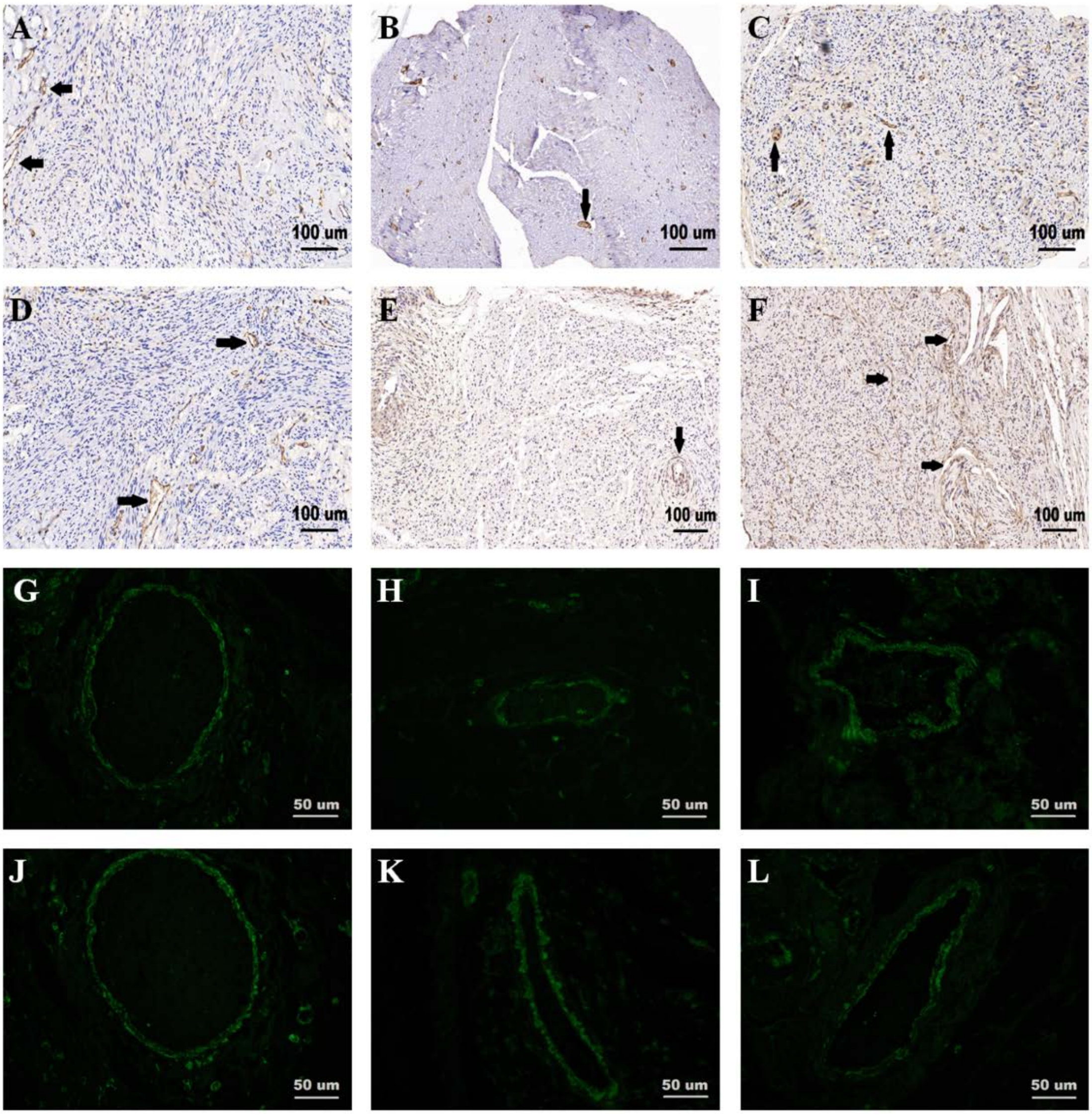

Figure S6. Assessment of angiogenesis in sciatic nerve regeneration. Immunohistochemistry staining for CD31 in regenerated nerve samples from the GO/PCL conduit (A and D), PCL conduit (B and E) and autograft (C and F) groups at 6 (A, B and C) and 12 weeks (D, E and F) after surgery. $CD31^+$ cells are indicated by arrows in each picture. Immunofluorescence staining for CD34 in regenerated nerve samples is also displayed for the GO/PCL conduit (G and J), PCL conduit (H and K) and autograft (I and L) groups at 6 (G, H and I) and 12 weeks (J, K and L) after surgery. The scale bars are 100 μm (A-F) and 50 μm (G-L), respectively.

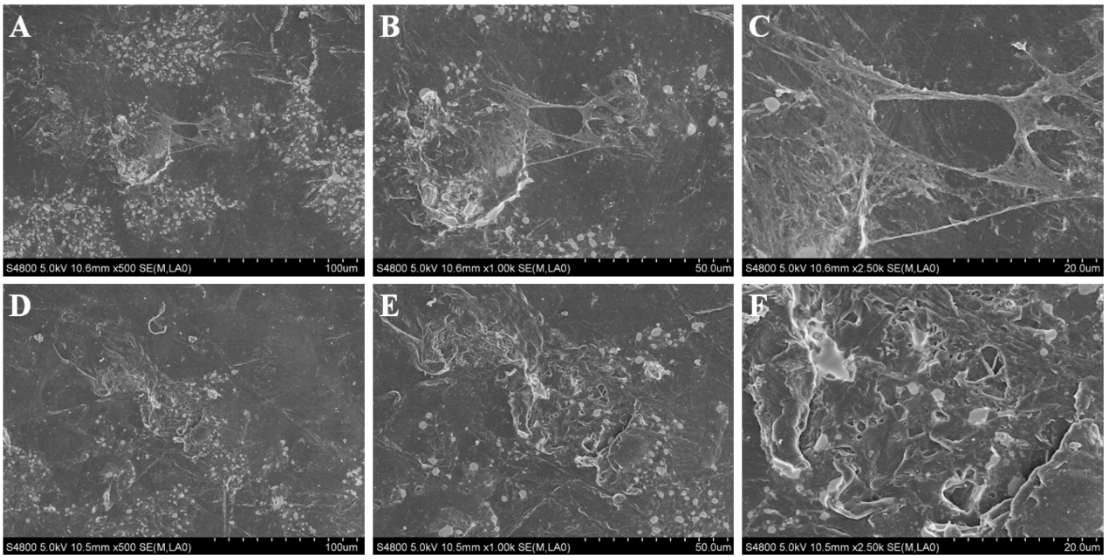

Figure S7. SEM showing cross-sectional pictures of RSCs seeded within GO/PCL (A-C) and PCL (D-F) scaffolds.

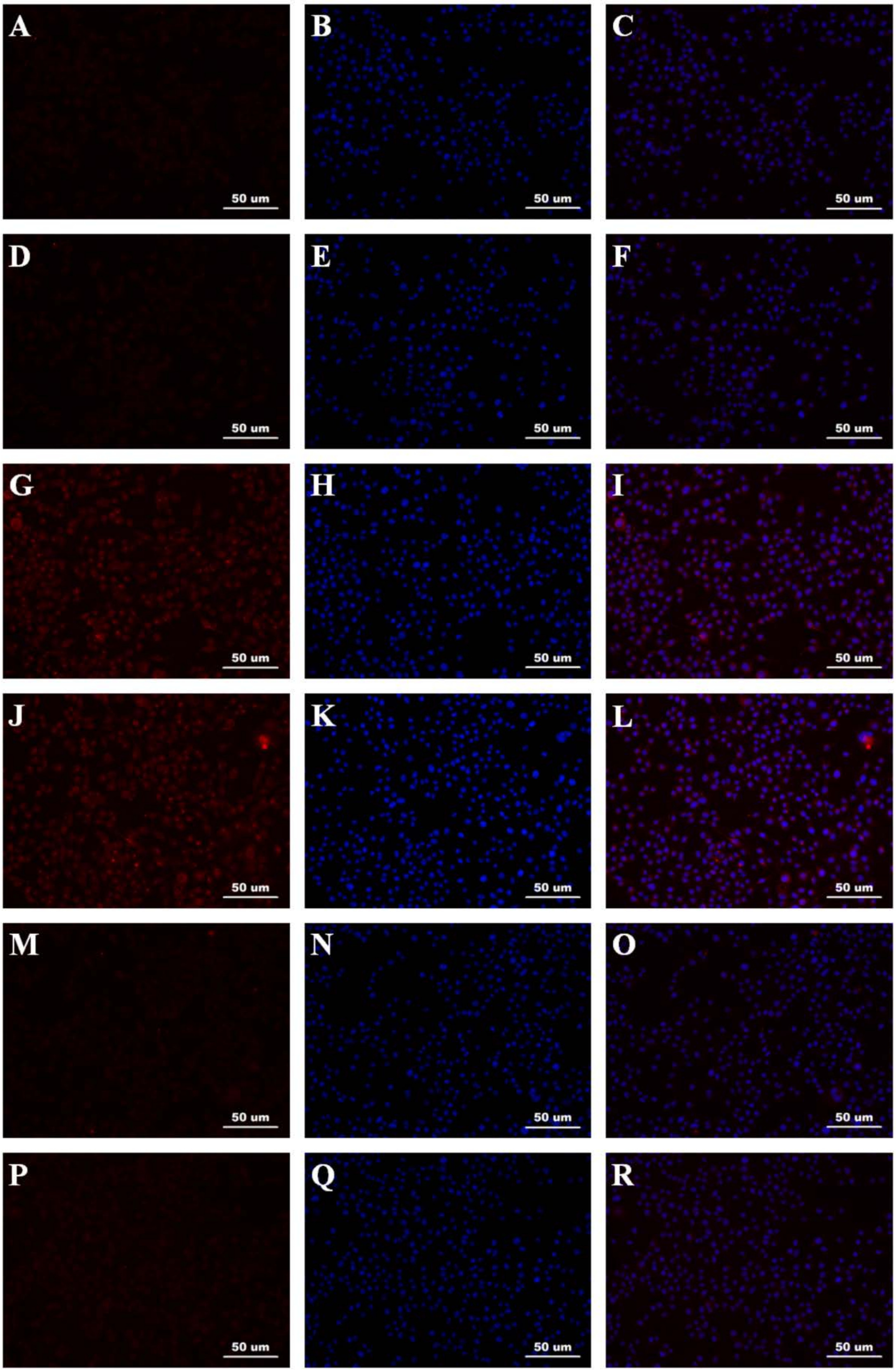

Figure S8. Immunofluorescence staining for GFAP (A-F), S100 (G-L) and nestin (M-R). All samples were washed three times, fixed with 4% paraformaldehyde for 20 minutes at 25°C and were blocked with BSA overnight. DAPI staining appears blue. PLGA (A-C, G-I and M-O). Collagen (D-F, J-L and P-R). The scale bar is 50 μm.

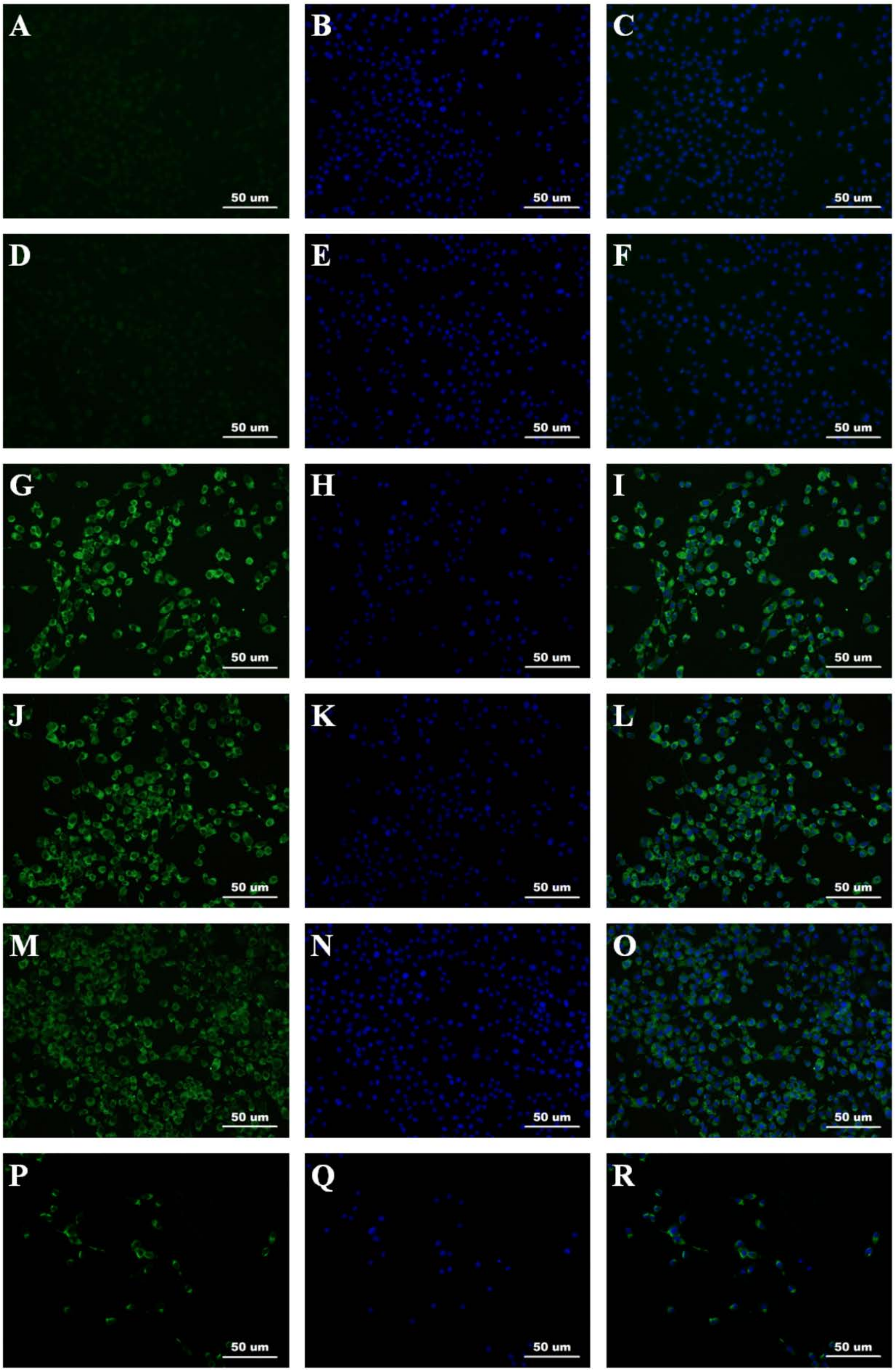

Figure S9. Immunofluorescence staining for Ki67 (A-F), and VEGF (G-R). All samples were washed three times, fixed with 4% paraformaldehyde for 20 minutes at 25°C and were blocked with BSA overnight. DAPI staining appears blue. GO/PCL (G-I). PCL (J-L), PLGA (A-C and M-O) and collagen (D-F and P-R). The scale bar is 50 μm.

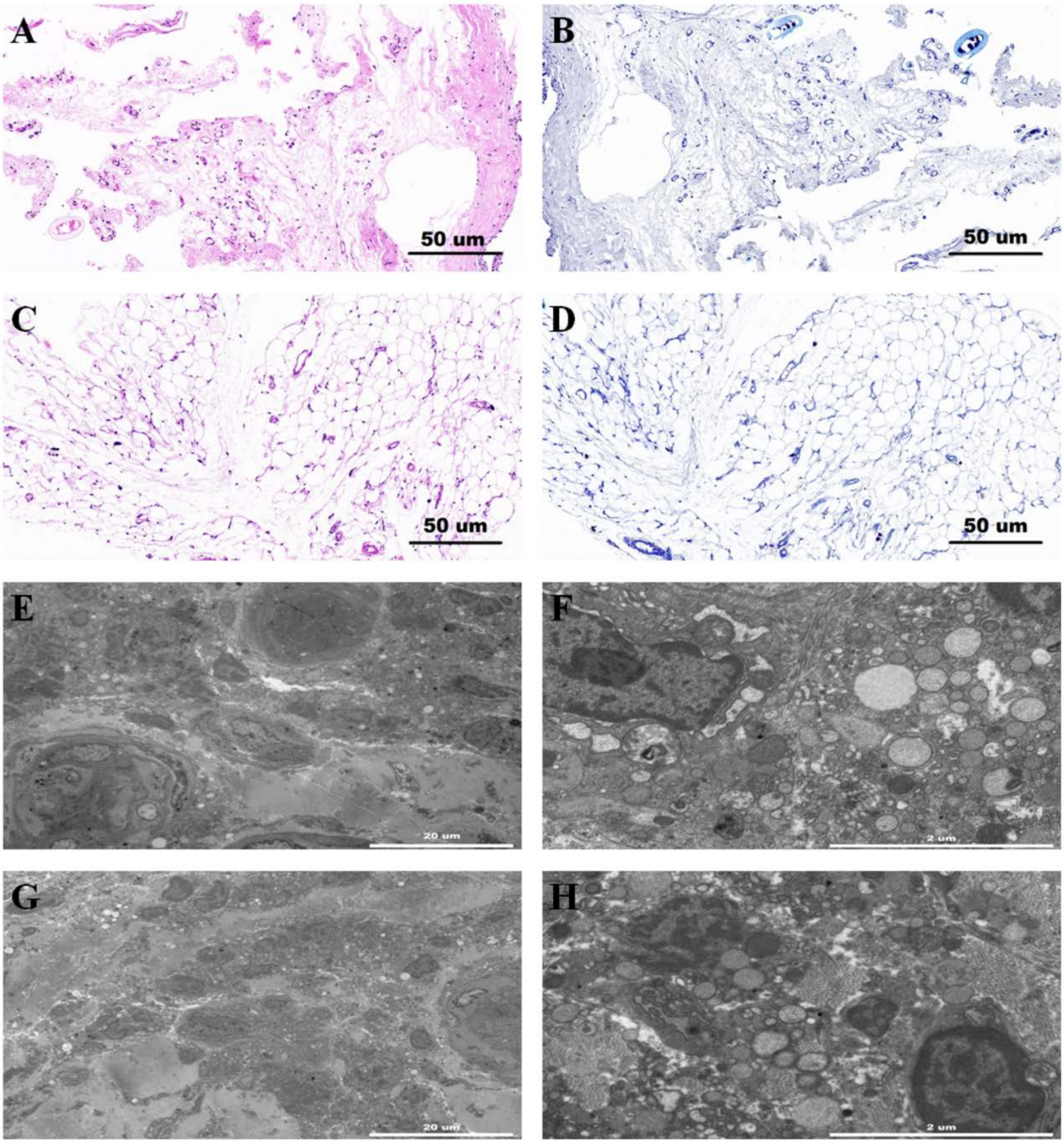

Figure S10. HE (A, C), TB (B, D) staining and TEM images (E-H) for 2% (A and B, E and F) and 4% (C and D, G and H) GO/PCL scaffolds at 18 weeks after surgery.

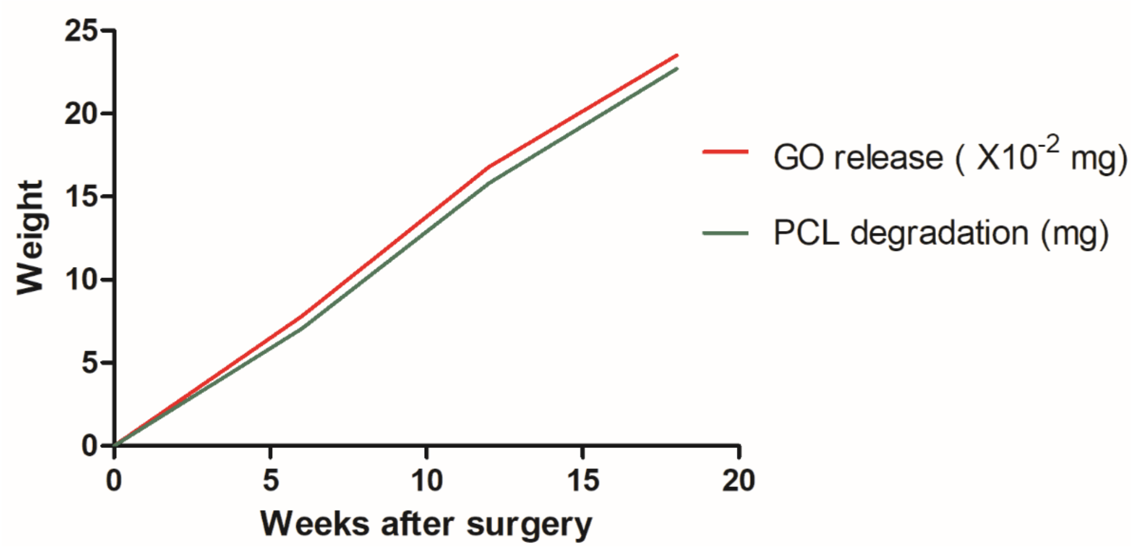

Figure S11. GO release and PCL degradation at 6, 12, 18 weeks after surgery and preoperatively.

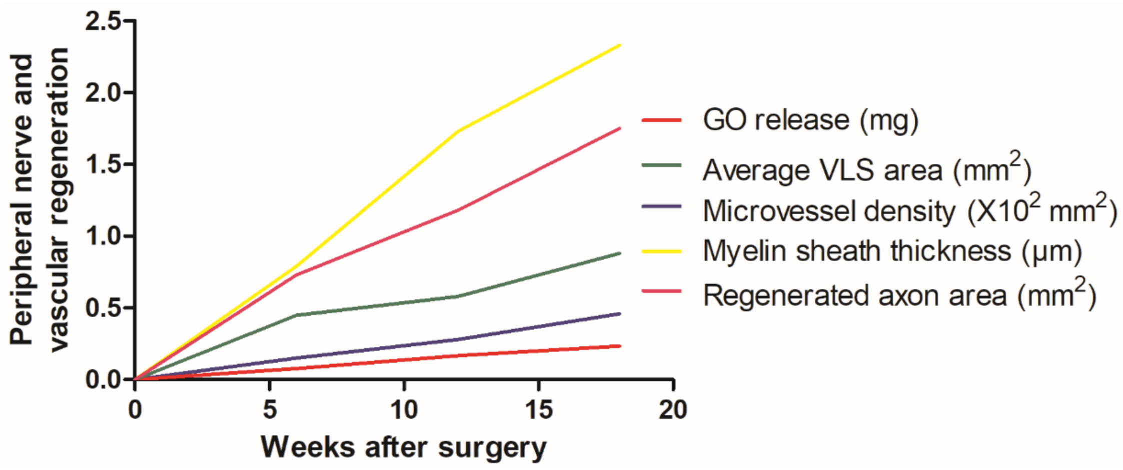

Figure S12. GO release and peripheral nerve and vascular regeneration at 6, 12, 18 weeks after surgery.

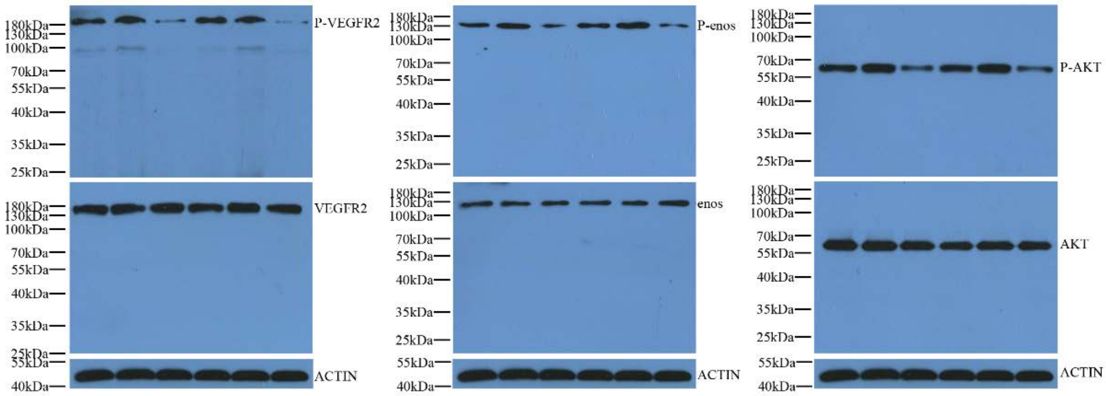

Figure S13. Full blots of AKT, p-AKT, eNOS, p-eNOS, VEGFR and p-VEGFR expression in regenerated nerves from the GO/PCL conduit, PCL conduit and autograft groups at 18 weeks after surgery. From left to right: GO/PCL conduit, autograft, PCL conduit, GO/PCL conduit, autograft and PCL conduit.

	Scaffold thickness (mm)	Elongation at break (%)	Elastic modulus (Mpa)
GO/PCL (ML)	0.46	42.3	48.32
PCL (ML)	0.45	34.2	31.77
GO/PCL (NL)	0.40	33.6	30.28
PCL (NL)	0.38	26.4	22.75

Table S1. Mechanical properties of 3D printing multi-layered GO/PCL and PCL scaffolds as well as non-layered GO/PCL and PCL scaffolds. ML, multi-layered; NL, non-layered.

Weeks after surgery	Remaining conduit (mg)	GO release ($X10^{-2}$ mg)	PCL degradation (mg)
0	34.600	0.000	0.000
6	27.460	7.800	7.062
12	18.600	16.800	15.832
18	11.670	23.500	22.695

Table S2. GO release and PCL degradation from GO/PCL conduit in long-term *in vivo* study.

Video S1. Walking track analysis of rats in the GO/PCL conduit, PCL conduit and autograft groups at 6, 12 and 18 weeks.
